# Mechanisms of MEOX1 and MEOX2 Regulation of the Cyclin Dependent Kinase Inhibitors p21^CIP1/WAF1^ and p16^INK4a^ in Vascular Endothelial Cells

**DOI:** 10.1371/journal.pone.0029099

**Published:** 2011-12-20

**Authors:** Josette M. Douville, David Y. C. Cheung, Krista L. Herbert, Teri Moffatt, Jeffrey T. Wigle

**Affiliations:** 1 Institute of Cardiovascular Sciences, St. Boniface Hospital Research Centre, Winnipeg, Manitoba, Canada; 2 Department of Biochemistry and Medical Genetics, University of Manitoba, Winnipeg, Manitoba, Canada; Katholieke Universiteit Leuven, Belgium

## Abstract

Senescence, the state of permanent cell cycle arrest, has been associated with endothelial cell dysfunction and atherosclerosis. The cyclin dependent kinase inhibitors p21^CIP1/WAF1^ and p16^INK4a^ govern the G_1_/S cell cycle checkpoint and are essential for determining whether a cell enters into an arrested state. The homeodomain transcription factor MEOX2 is an important regulator of vascular cell proliferation and is a direct transcriptional activator of both p21^CIP1/WAF1^ and p16^INK4a^. MEOX1 and MEOX2 have been shown to be partially functionally redundant during development, suggesting that they regulate similar target genes *in vivo*. We compared the ability of MEOX1 and MEOX2 to activate p21^CIP1/WAF1^ and p16^INK4a^ expression and induce endothelial cell cycle arrest. Our results demonstrate for the first time that MEOX1 regulates the MEOX2 target genes p21^CIP1/WAF1^ and p16^INK4a^. In addition, increased expression of either of the MEOX homeodomain transcription factors leads to cell cycle arrest and endothelial cell senescence. Furthermore, we show that the mechanism of transcriptional activation of these cyclin dependent kinase inhibitor genes by MEOX1 and MEOX2 is distinct. MEOX1 and MEOX2 activate p16^INK4a^ in a DNA binding dependent manner, whereas they induce p21^CIP1/WAF1^ in a DNA binding independent manner.

## Introduction

The G_1_/S cell cycle checkpoint is critical for determining whether a cell will enter into S phase and replicate its genome, or enter into an arrested state and delay cellular proliferation [1]. This state of arrest can be either temporary (quiescence) or it can be permanent (senescence) [1]. Cells of the adult vasculature remain in the quiescent state until a need for new blood vessels is encountered, such as in wound healing [2], menstruation [3] and exercise [4]. Aged blood vessels, however, have impaired angiogenic capabilities [5].

Senescence is associated with aging and is also thought to contribute to atherosclerotic vascular disease [6], [7]. It has been shown in humans that blood vessels with atherosclerotic plaques contain a higher proportion of senescent vascular smooth muscle and endothelial cells, as compared to non-atherosclerotic vessels [8]–[10]. Senescent endothelial cells have reduced nitric oxide synthase expression [11]–[13] and altered metabolism, which results in endothelial dysfunction and contributes to the progression of vascular diseases, such as atherosclerosis [6], [7].

The cyclin dependent kinase inhibitors (CKIs) p21^CIP1/WAF1^ and p16^INK4a^ block cellular proliferation and govern the G_1_/S cell cycle checkpoint [14]. These CKIs bind to cyclin dependent kinases (CDK2 and/or CDK4), impeding their association with cyclin proteins (Cyclin E or D, respectively) and thereby prevent activation of the CDKs. In the absence of activated CDKs, the subsequent phosphorylation of the retinoblastoma protein, a requirement for cell cycle progression from G_1_ to S phase, does not occur [14]. The CKIs p21^CIP1/WAF1^ and p16^INK4a^ are encoded by the *CDKN1A* and *CDKN2A* genes, respectively and are currently the only confirmed transcriptional targets of Mesenchyme homeobox 2 (MEOX2) [15], [16].

Together, MEOX1 and MEOX2 comprise a family of diverged homeodomain transcription factor proteins. Both MEOX1 and MEOX2 are expressed in all cells of the adult cardiovasculature, including cardiomyocytes [17]–[19], cardiac fibroblasts [17], vascular smooth muscle and endothelial cells [18], [20]–[22]. The role of MEOX2 in the vasculature has been extensively studied, however very little is known about the role of MEOX1 in this system. MEOX1 has been shown to be expressed in blood, but not lymphatic, endothelial cells [23] and ectopic MEOX1 expression induces the cardiomyogenic differentiation of P19 pluripotent embryonic carcinoma cells [24].

MEOX2 has been shown to prevent vascular cell proliferation by mediating the transcriptional up-regulation of the p21^CIP1/WAF1^ gene and ectopic expression of MEOX2 prevents blood vessel stenosis [25], [26]. MEOX2 expression is diminished in vascular smooth muscle cells after *in vitro* mitogen stimulation [27] and in mechanically injured endothelial cells *in vivo* [28], thereby permitting vascular cell proliferation. Likewise blood vessels from individuals affected by hepatic portal hypertension have lower levels of MEOX2 expression when compared to non-affected individuals [29], presumably as a response to vessel injury due to increased blood pressure.

MEOX1 and MEOX2 are necessary for proper bone and skeletal muscle formation in the developing mouse embryo [30]. Knockout of both MEOX1 and MEOX2 in mice produces a phenotype that is more severe than the predicted additive phenotypes of the single MEOX gene knockout mice [30]. This finding suggests that MEOX1 and MEOX2 have overlapping functions during embryonic development. Therefore, it is likely that MEOX1 and MEOX2 share transcriptional target genes. MEOX1 was shown to function as a direct transcriptional activator of the NKX3-2/BAPX1 gene by binding to the NKX3-2 promoter and increasing its expression [31]. Similarly, MEOX2 was able to bind to the same AT rich consensus site within the NKX3-2 promoter [31]. We therefore hypothesized that like MEOX2, MEOX1 would activate the transcription of p21^CIP1/WAF1^ and p16^INK4a^ in endothelial cells.

In the present study, we investigated the role of MEOX1 and MEOX2 in controlling endothelial proliferation and senescence, as well as the mechanisms of transcriptional activation of p21^CIP1/WAF1^ and p16^INK4a^ by the MEOX transcription factors. We demonstrate for the first time that MEOX1 activates both p21^CIP1/WAF1^ and p16^INK4a^ expression in endothelial cells and that both MEOX1 and MEOX2 can effectively induce endothelial cell senescence. In addition, we show that MEOX1 and MEOX2 activation of p16^INK4a^ gene is dependent upon DNA binding whereas their activation of p21^CIP1/WAF1^ gene transcription is via a DNA binding independent mechanism.

## Materials and Methods

### MEOX1 and MEOX2 fusion protein expression constructs

Full-length mouse MEOX1 and human MEOX2 were amplified by PCR from cDNA clones (IMAGE ID 464899 and 3917118, Invitrogen) and cloned into the pCMV-Tag2B or pcDNA3.1 vectors to create either N-terminal or C-terminal FLAG tagged fusion proteins, respectively. The DNA binding deficient constructs MEOX1^Q220E^ and MEOX2^Q235E^ were created by PCR mutagenesis of the MEOX1 and MEOX2 constructs, which changed CAA (Q) to GAA (E) at amino acid position 220 and 235, respectively. The homeodomain deleted construct MEOX2^K195_K245del^ was created by splice overlap extension PCR [32] to create a MEOX2 construct lacking amino acids 195-245. All expression constructs were sequence verified. Additional details regarding the cloning of these constructs and primer sequences are listed in Table S1.

### Adenoviral constructs

Ad-EGFP was a gift from Dr. G. Pierce (University of Manitoba) and Ad-p53-EGFP was a gift from Dr. N. Mesaeli (Weill Cornell Medical College in Qatar). Adenovirus encoding N-terminal FLAG tagged MEOX1 and MEOX2 adenoviral constructs were created by excising MEOX1 and MEOX2 from the pCMV-Tag2B vector by *NotI/XhoI* digestion, followed by ligation into the pShuttle vector. Production and amplification of the adenoviral stocks was achieved using the AdEasy vector system (Qbiogene). Adenoviral titres were determined using the RapidTiter kit (Clontech).

### p21^CIP1/WAF1^ and p16^INK4a^ promoter luciferase constructs and expression vectors

The WWP-LUC vector, a gift from Dr. B. Vogelstein (Johns Hopkins University) [33], was digested with *SstI/HindIII* and the 2272 bp human p21^CIP1/WAF1^ promoter was cloned into the pGL3-basic vector (Promega). The 849 bp, 505 bp, 426 bp and 232 bp p21^CIP1/WAF1^ promoters were created using primers listed in Table S2. The 103 bp p21 promoters (p21P 93-S WT, MT1, MT2, MT3 and MT4 [34]) were a gift from Dr. B. Sawaya (Temple University) [35]. The pGL3 vector containing the 564 bp p16^INK4A^ promoter (pGL3-INK4a) was a gift from Dr. S. Chanda (Burnham Institute for Medical Research) [16]. The SP1 expression plasmid (pCMV4-Sp1/flu) was a gift from Dr. J. Horowitz (North Carolina State University) [36]. The pcDNA3-lacZ vector was a gift from Dr. N. Mesaeli (Weill Cornell Medical College in Qatar).

### Luciferase assays

HEK293 cells (ATCC) were cultured in HyQ DMEM/High Glucose (HyClone) containing 5% Fetal Bovine Serum (FBS) (HyClone) and 1% Penicillin/Streptomycin (Gibco). Human umbilical vein endothelial cells (HUVECs) (Clonetics) were cultured in EGM-2 (Clonetics). Media was changed to Opti-MEM I (Gibco) containing 10% Calf Serum (Gibco) 48 hours after plating 1.5×10^5^ cells/well. Cells were transfected using Lipofectamine 2000 or Lipofectamine LTX Reagent (Invitrogen). Additional details can be found in Methods S1. In all experiments, media was changed back to growth medium 4 hours post-transfection. Mithramycin A (200 ng/mL final concentration [37], [38]), or the same volume of methanol (vehicle), was diluted in growth medium and added to the cells 4 hours post-transfection.

Luciferase assays were performed 24 hours after transfection or mithramycin A treatment using a Lumat LB 9507 luminometer and luciferase buffer containing 20 mM Tricine, 1.07 mM MgCO_3_, 2.67 mM MgSO_4_, 0.1 mM EDTA, 33.3 mM DTT, 270 µM coenzyme A, 470 µM luciferin, and 530 µM ATP. β-galactosidase assays were performed using a solution containing 0.801 µg/µL ONPG and a MRX-TC revelation spectrophotometer (Dynex Technologies) set to 415 nm. To control for transfection efficiency, luciferase assay values were normalized to the β-galactosidase assay values for each sample. Empty expression vectors were used to control for basal promoter activity. Fold activation was calculated by dividing the relative luciferase unit value of each sample by the value obtained for the empty vector control.

### Immunofluorescence

HUVECs (1×10^5^ cells/plate) were transduced at a multiplicity of infection (MOI) of 250 with adenovirus and then plated onto collagen I (BD Biosciences) coated glass coverslips. Forty-eight hours after transduction, cells were fixed in 4% paraformaldehyde (EMD Chemicals) and then blocked with 5% goat serum (Sigma) in PBS containing 0.3% Triton-X 100 (PBS-T). Coverslips were incubated with primary mouse anti-FLAG antibody [M2] (Sigma) followed by Alexa Fluor 488 conjugated goat anti-mouse IgG secondary antibody (Invitrogen). Subsequently, coverslips were incubated with propidium iodide (Invitrogen). The coverslips were then mounted onto slides using FluorSave Reagent (CalBiochem). Images were acquired with an Olympus IX70 confocal laser microscope using FluoView 2.0 software. Additional details can be found in Methods S1.

### Western blotting and quantification

HEK293 cells were cultured as described above. Forty-eight hours after plating 2×10^5^ cells/6 cm tissue culture plate, the media was changed to Opti-MEM I (Gibco) containing 10% Calf Serum (Gibco). Each plate of cells was then transfected with 4μg MEOX expression vector DNA using 10 µL Lipofectamine 2000 Reagent (Invitrogen). Media was changed back to growth medium after 4 hours and cells were harvested 48 hours post-transfection. 1×10^5^ trypsinized HUVECs were transduced at 250 MOI with adenovirus and then plated onto 6 cm tissue culture plates. Cells were harvested 48–72 hours post-transduction.

For whole cell lysates, cells were harvested using RIPA buffer (50mM Tris pH 7.4, 150mM NaCl, 1mM EDTA, 1mM EGTA, 0.5% Na-deoxycholate, 1% Triton-X 100 and 0.1% SDS) containing complete mini protease inhibitor cocktail (Roche). Whole cell lysates were centrifuged for 15 seconds to pellet cell debris. To assure equal loading between samples, protein assays were performed prior to sample preparation using the DC Protein Assay Kit (Bio-Rad) and an Ultrospec 2000 (Pharmacia Biotech) or MRX-TC revelation spectrophotometer (Dynex Technologies) set to 540 nm. Nuclear and cytosolic proteins were isolated using the NE-PER nuclear and cytoplasmic extraction kit (Pierce). All samples were prepared with 3× loading buffer (166.4 mM Tris pH 7.4, 33.3% glycerol, 6.6% SDS, 0.3% bromophenol blue and 100 mM DTT) and then boiled for 5 minutes to denature the proteins prior to loading.

Proteins were separated by SDS-PAGE and then transferred to nitrocellulose membranes (Bio-Rad). Primary antibodies used for western blotting were: mouse anti-FLAG antibody [M2] (Sigma), mouse anti-α-tubulin antibody [DM1A] (Abcam), mouse anti-p21^CIP1/WAF1^ antibody [CP74] (Sigma), rabbit anti-actin antibody (Sigma) and mouse anti-p16^INK4a^ antibody [DCS-50] (Santa Cruz). A full description of blotting conditions can be found in Methods S1.

### Quantitative real-time PCR

Real-time PCR experiments were performed using RNA from HUVECs (2.5×10^5^ cells/plate) transduced at 250 MOI with adenovirus for 48–72 hours. RNA was isolated using the RNeasy Plus kit (Qiagen) following the manufacturer's instructions. One-step real-time PCR was performed using an iQ5 thermocycler (BioRad) and the iScript One-Step PCR kit with SybrGreen (BioRad) or the BR 1-Step SYBR Green qRT-PCR Kit (Quanta). Relative gene quantification (2^-ΔΔCT^ method) was performed where the mRNA expression of p21^CIP1/WAF1^ and p16^INK4a^ was compared to the mRNA expression of the β-actin control. PCR products were resolved on 2% agarose gels and cloned using the TOPO TA cloning kit (Invitrogen). The resulting constructs were verified by sequencing. Primer sequences are listed in Table S3.

### Electrophoretic mobility shift assays (EMSA)

Details about recombinant protein production can be found in Methods S1. Two hundred nanograms of recombinant GST-fusion protein was used per EMSA binding reaction. Alternatively, HUVECs were transduced at a MOI of 50 with adenovirus then seeded onto tissue culture plates. Nuclear proteins were isolated using the NE-PER nuclear and cytoplasmic extraction kit (Pierce) 72 hours post-transduction and 5 µL nuclear extract was used per binding reaction.

EMSAs were carried out using the LightShift Chemiluminescent EMSA Kit (Pierce). The sequence of the EMSA probes containing either the distal or the proximal homeodomain binding sites from the p16^INK4a^ promoter are listed in Table S4. Binding reactions (20 µL) were incubated for 30 minutes at room temperature in a buffer containing 10 mM Tris pH 7.5, 50 mM KCl, 1 mM DTT, 50 ng/μl Poly(dI•dC), 5% glycerol, 0.05% NP-40, 3.5mM MgCl_2_, 0.5 mM EDTA, 0.25 mg/ml BSA and 30 – 90 fmol biotin end-labelled probe. For cold competition reactions, 8 – 18 pmol unlabelled probe was added (200 molar excess) and then incubated for 15 minutes at room temperature prior to the addition of the biotin labelled probe. Super-shift reactions containing 1 – 1.5 µg normal mouse IgG (Millipore) or anti-FLAG [M2] antibody (Sigma) were incubated overnight at 4°C, prior to the addition of biotin labelled probe. Luminescence was detected using CL-Xposure blue X-ray film (Thermo Scientific).

### Senescence associated β-gal assays

HUVECs (1×10^5^ cells/well) were transduced with adenovirus at 250 MOI and then plated onto collagen I (BD Biosciences) coated glass coverslips. Forty-eight hours post-transduction, cells were washed twice with PBS and then fixed for 5 minutes at room temperature with 2% paraformaldehyde (EMD Chemicals) diluted in PBS. Coverslips were washed twice with PBS and then freshly prepared SA-β-gal staining solution (40 mM citric acid/sodium phosphate, pH 6.0, 150 mM NaCl, 2 mM MgCl_2_, 5 mM potassium ferricyanide, 5 mM potassium ferrocyanide, 1 mg/mL X-gal) was added and incubated overnight at 37°C [39]. The following day, coverslips were rinsed three times with double distilled water. Nuclei were stained with Mayer's hematoxylin solution (Sigma) at room temperature for 2 minutes and then rinsed three times with double distilled water. Coverslips were mounted onto glass slides using FluorSave Reagent (CalBiochem). Phase contrast images of 16 random fields (20×) per coverslip were acquired using a Zeiss Axioskop 2 mot plus microscope equipped with an AxioCam digital camera and AxioVision 4.6 software (Zeiss). The number of SA-β-gal positive cells was counted by an observer that was blinded to the identity of the slides and then expressed as a percentage of the total number of cells counted.

### Cell cycle analysis

HUVECs (3×10^5^ cells/plate) were transduced with adenovirus at 100 MOI and then plated. Forty-eight hours post-transduction cells were treated with 5′-bromo-2′-deoxyuridine (BrdU) (Fisher) at a final concentration of 10 µM for 1 hour at 37°C + 5% CO_2_. Cells were washed three times with PBS, trypsinized and then pelleted by centrifugation. The cell pellets were resuspended in 0.5 mL PBS, following which 2 mL cold 70% ethanol was added and the cells were fixed overnight at 4°C. Cells were pelleted by centrifugation and then resuspended in 1 mL freshly prepared 2N HCl and incubated at room temperature for 25 minutes. Subsequently, 2 mL PBS containing 3% FBS (PBS/FBS) was added to the cells, which were then pelleted by centrifugation. The cell pellet was resuspended in 1 mL 0.1 M sodium borate pH 8.5 and incubated at room temperature for 2 minutes. PBS/FBS (2 mL) was added to the cells, which were then pelleted by centrifugation. This step was repeated, following which cells were resuspended in 0.1 mL PBS/FBS containing 5 μL Alexa Fluor 488 conjugated mouse anti-BrdU [MoBU-1] antibody and then incubated for 2 hours at room temperature. Subsequently, 2 mL PBS/FBS was added to the cells, which were then pelleted by centrifugation. Lastly, the cell pellet was then resuspended in 0.5 mL PBS/FBS containing 10 μL 0.2 mg/mL 7-aminoactinomycin D (7-AAD) (Invitrogen) and incubated for 15 minutes at room temperature. All centrifugation steps were carried out at 350 × g for 5 minutes at room temperature. 1×10^4^ gated cells per sample were counted using a BD FACSCalibur flow cytometer. The results were analyzed using FlowJo software (Tree Star, Inc.).

### Apoptosis assays

HUVECs (1×10^5^ cells/well) were transduced with adenovirus at 250 MOI and then plated onto collagen I (BD Biosciences) coated glass coverslips. Prior to fixation, one plate of HUVECs was treated with staurosporine (Fisher) at a final concentration of 2.5 µM for 4 hours at 37°C + 5% CO_2_. 48 hours post-transduction, cells were washed once with PBS and then fixed for 30 minutes at room temperature with 4% paraformaldehyde (EMD Chemicals). The coverslips were washed three times with PBS-T and then terminal deoxyuridine nick end labelled (TUNEL) using the *In Situ* Cell Death Detection Kit with TMR red (Roche). Briefly, the washed coverslips were incubated with the TUNEL reaction mixture for 60 minutes at room temperature, rinsed three times with PBS and then mounted onto glass slides using SlowFade Gold antifade reagent with DAPI (Invitrogen). Fluorescence images of 16 random fields (20×) per coverslip were acquired using a Zeiss Axioskop 2 mot plus microscope. The number of TUNEL positive nuclei was counted by an observer that was blinded to the identity of the slides and then expressed as a percentage of the total number of nuclei counted.

### Statistical analysis

Two-Sample t-tests were used to evaluate the changes between MEOX proteins and the empty vector as well as to compare the effect of MEOX1 to that of MEOX2 for luciferase assay data. Pair-Sample T-tests were used to evaluate the changes between MEOX proteins and the EGFP control as well as to compare the effect of MEOX1 to that of MEOX2 for all other experiments. Changes were considered significant if the p-value was less than 0.05. Statistical analysis was performed using Origin 8.5 software.

## Results

### Expression and subcellular localization of the MEOX proteins

**Figure 1:**
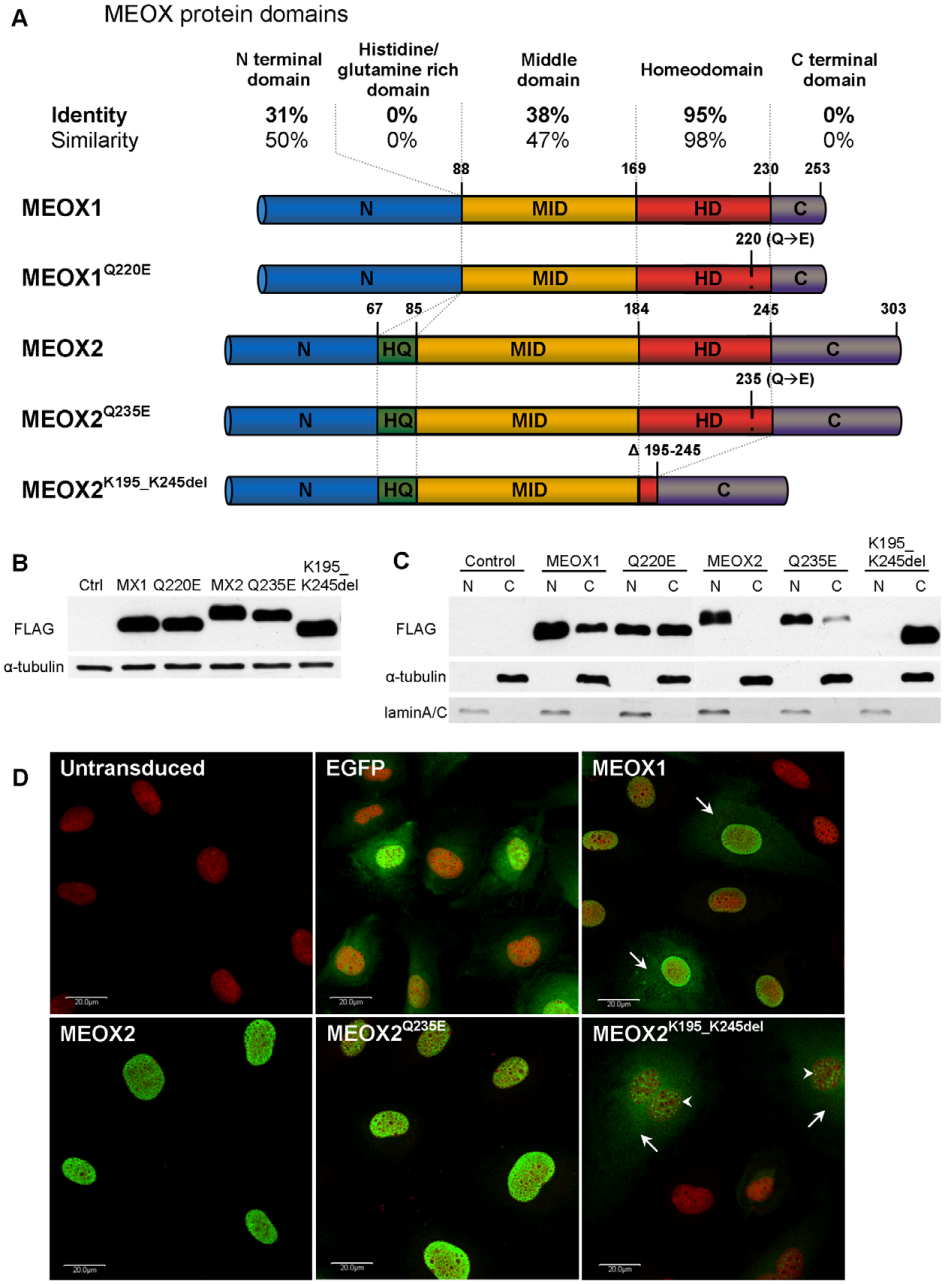
Expression and subcellular localization of MEOX1 and MEOX2 proteins. A) Schematic representation of the MEOX1 and MEOX2 protein constructs used in this paper. The alignment lists the percentage of similar and identical amino acids conserved between human MEOX1 and MEOX2 in the different functional protein domains. B) Representative western blot displaying the relative level of MEOX1 (MX1), MEOX2 (MX2), DNA binding deficient MEOX1^Q220E^ (Q220E), DNA binding deficient MEOX2^Q235E^ (Q235E) and homeodomain deleted MEOX2^K195_K245del^ (K195_K245del) protein expression in HEK293 cells 48 hours after transfection. The N-terminally tagged MEOX proteins were detected using an anti-FLAG antibody and α-tubulin was used as a loading control. The empty expression vector was used as a negative control (Ctrl). C) A representative western blot demonstrating the subcellular localization of different MEOX proteins in HEK293 cells, 48 hours after transfection. α-tubulin was used as a cytoplasmic (C) marker and lamin A/C was used as nuclear (N) marker. D) Representative fluorescent immunocytochemistry showing the localization and level of expression of the MEOX proteins in HUVECs 48 hours after adenoviral transduction at a multiplicity of infection of 250. The N-terminally tagged MEOX proteins were detected using an anti-FLAG antibody (green) and nuclei were stained with propidium iodide (red). Enhanced green fluorescent protein (EGFP) was used as a control for adenoviral infection. Arrows indicate cytoplasmic staining and arrowheads indicate punctate nuclear aggregates. Scale bar represents 20 µm.

We analyzed the degree of conservation between the various functional domains of MEOX1 and MEOX2 (Figure 1A). While the MEOX homeodomains (HD) are nearly identical (95%), there is only a low degree of homology between MEOX1 and MEOX2 outside of this domain. The N-terminus (N) and middle domain (MID) are the next most conserved regions of these proteins, with only 35% and 38% amino acid identity, respectively. Unlike MEOX2, MEOX1 does not contain a histidine/glutamine (HQ) rich domain, which is a putative transactivation domain [15].

MEOX1 and MEOX2 expression constructs (Figure 1A) were generated with a FLAG epitope at either the N-terminus or the C-terminus of the protein. In addition to wild-type MEOX1 and MEOX2, we created constructs in which the homeodomains were mutated in order to study the DNA binding requirement of MEOX protein function. The MEOX1^Q220E^ and the MEOX2^Q235E^ constructs contain the entire MEOX homeodomain, but include a glutamine to glutamate substitution at position 50 of the homeodomain (Figure 1A). This mutation has previously been shown to result in DNA binding defective homeodomain proteins [40], [41]. As well, a deletion version of MEOX2 was generated, MEOX2^K195_K245del^, which lacks nearly the entire homeodomain (Figure 1A).

The level of expression and localization of the various FLAG tagged MEOX proteins were verified by western blot and fluorescent immunocytochemistry. Comparable levels of protein expression were observed by western blot of MEOX transfected HEK293 cell lysates (Figure 1B; Figure S1, panel A) and Ad-MEOX transduced HUVEC lysates (Figure S1, panel B). MEOX1 and MEOX2 were localized predominantly to the nucleus, although MEOX1 was also consistently detected in the cytoplasm (Figure 1C, 1D). MEOX1^Q220E^ and MEOX2^Q235E^ were both localized to the nucleus, however an increased amount of cytosolic protein was detected for both mutants as compared to the wild-type MEOX proteins (Figure 1C). In contrast, MEOX2^K195_K245del^ differed dramatically in localization from that of either the wild-type MEOX2 or the MEOX2^Q235E^ proteins, as it was detected predominantly in the cytoplasm and also as punctate nuclear aggregates (Figure 1C, 1D). Further use of this construct was therefore halted, given this confounding factor of altered subcellular localization.

The FLAG epitope did not affect the expression of the MEOX proteins, as similar levels of expression and subcellular localization were obtained for N- and C-terminally tagged constructs (data not shown). Likewise, luciferase assays performed with N-, C- and non-tagged versions of MEOX2 confirmed that the FLAG epitope does not affect the ability of MEOX2 to induce transcription (Figure S2).

### MEOX1 activates p21^CIP1/WAF1^ expression in endothelial cells

**Figure 2:**
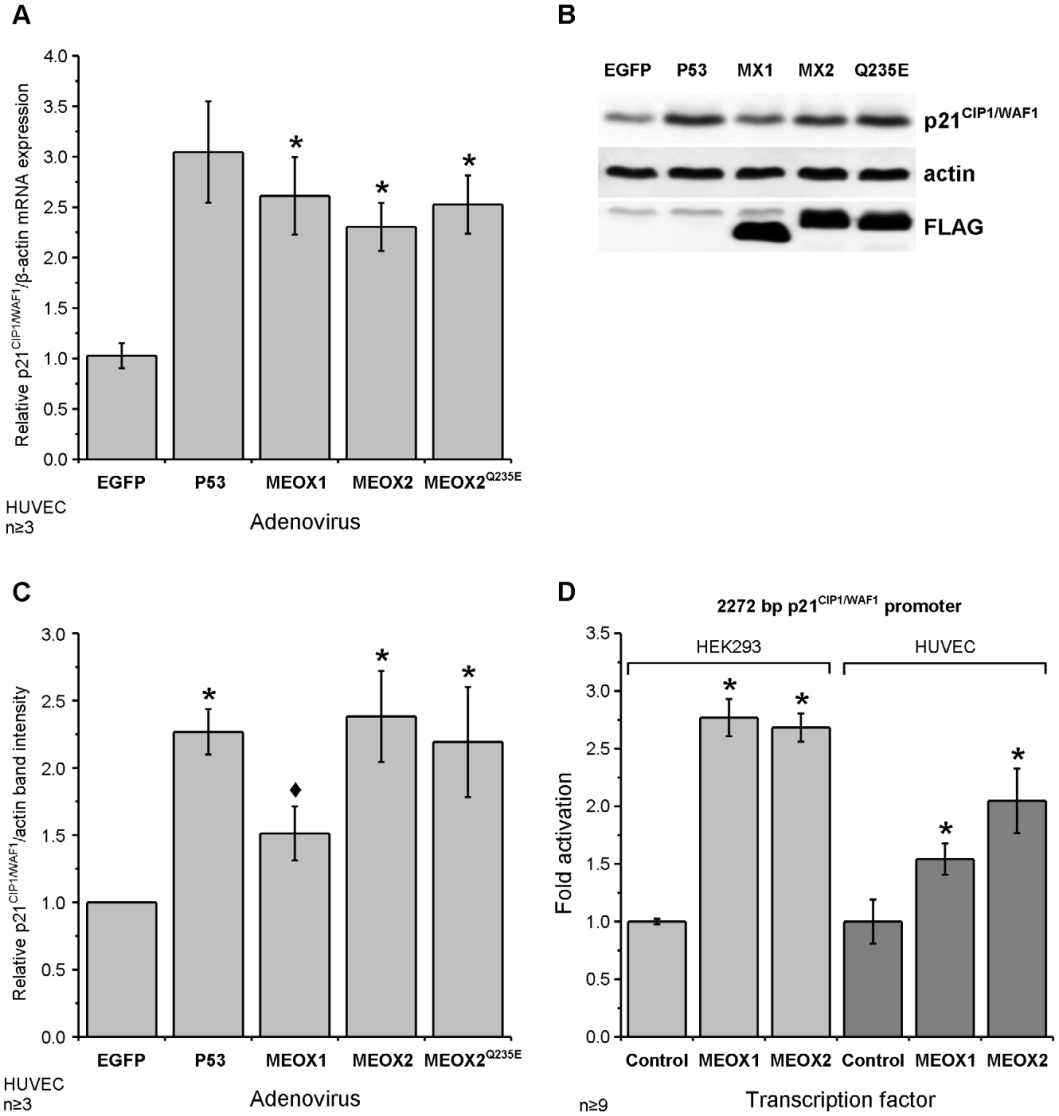
MEOX1 and MEOX2 activate expression of p21^CIP1/WAF1^ mRNA and protein in endothelial cells. A) Relative level of endogenous p21^CIP1/WAF1^ mRNA compared to EGFP transduced HUVECs. Total RNA was isolated from HUVECs 48 hours after adenoviral transduction at a multiplicity of infection (MOI) of 250 and the amount of p21^CIP1/WAF1^ mRNA was measured by quantitative real-time PCR. Beta-actin mRNA expression was used for inter-sample normalisation. B) A representative western blot showing increased p21^CIP1/WAF1^ protein in HUVECs expressing ectopic MEOX2 (MX2) or DNA binding mutant MEOX2^Q235E^ (Q235E) but not MEOX1 (MX1). Total protein was isolated from HUVECs 48 hours after adenoviral transduction at 250 MOI. C) Quantification of the relative amount of p21^CIP1/WAF1^ protein compared to EGFP transduced HUVECs. The intensity of the p21^CIP1/WAF1^ band was normalized to the actin loading control. D) Activation of the 2272 bp p21^CIP1/WAF1^ promoter driven luciferase reporter gene by MEOX1 and MEOX2 in HEK293 and HUVECs. * Indicates a statistically significant change (p<0.05) when compared to the empty vector or EGFP control. ♦ Indicates a statistically significant difference (p<0.05) between MEOX1 and MEOX2.

To assess the ability of MEOX1 and MEOX2 to induce the expression of endogenous p21^CIP1/WAF1^ in endothelial cells, we transduced HUVECs using adenoviral vectors, following which we performed quantitative real-time PCR and western blot analysis to measure the changes in p21^CIP1/WAF1^ expression at the mRNA and protein level, respectively. Ectopic expression of p53 was used as a positive control for the induction of p21, since p53 is a well characterized transcriptional activator of the p21^CIP1/WAF1^ gene [33], [42]. Compared to the EGFP control, we observed a three-fold increase in p21^CIP1/WAF1^ mRNA levels and more than a two-fold increase in p21^CIP1/WAF1^ protein levels 48 hours after adenoviral delivery of p53 (Figure 2A, 2C). Likewise, expression of MEOX1 or MEOX2 resulted in significantly increased p21^CIP1/WAF1^ mRNA expression. Interestingly, although MEOX1 was as effective as MEOX2 at inducing p21^CIP1/WAF1^ mRNA expression (Figure 2A), this potency did not correlate with a similar induction of p21^CIP1/WAF1^ protein (Figure 2B, 2C). MEOX2 increased p21^CIP1/WAF1^ protein expression comparable to p53, however, MEOX1 was a significantly less potent inducer of p21^CIP1/WAF1^ protein as compared to MEOX2 (Figure 2C). Next, we tested the ability of MEOX1 to activate transcription of a p21^CIP1/WAF1^ reporter gene. Comparable to MEOX2, MEOX1 activated the expression of a luciferase reporter from the 2272 bp p21^CIP1/WAF1^ promoter in HEK293 cells (Figure 2D). This activation was also seen in HUVECs (Figure 2D).

### MEOX1 activates p16^INK4a^ expression in endothelial cells

**Figure 3:**
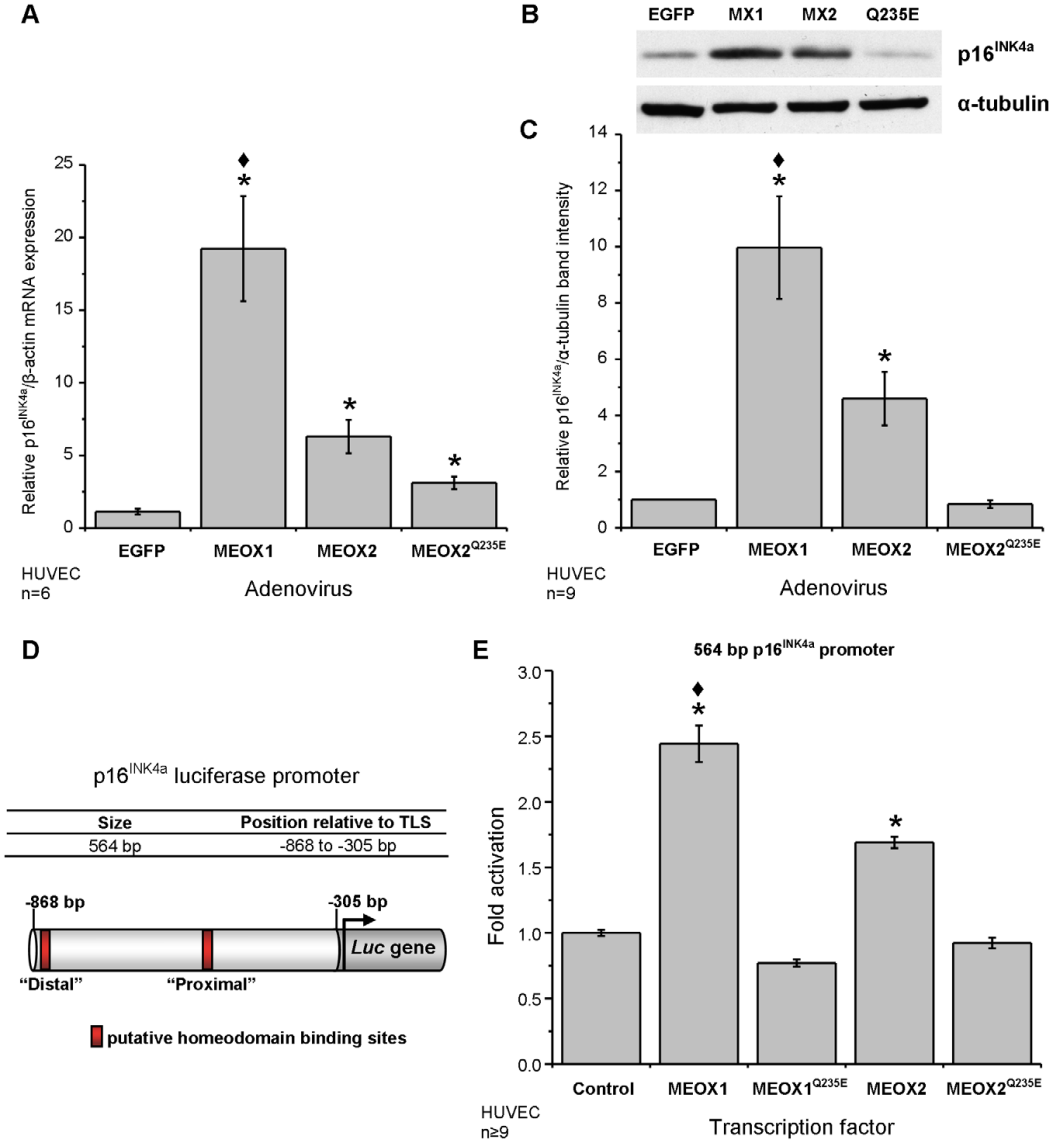
MEOX1 and MEOX2 activate transcription of the p16^INK4a^ gene in HUVECs. A) Relative level of p16^INK4a^ mRNA in HUVECs. Total RNA was isolated from HUVECs 72 hours after adenoviral transduction at a multiplicity of infection (MOI) of 250 and the amount of p16^INK4a^ mRNA was measured by quantitative real-time PCR and compared to EGFP transduced HUVECs. Beta-actin mRNA expression was used for inter-sample normalisation. MEOX1 is a more potent activator of p16^INK4a^ than MEOX2. B) A representative western blot showing increased p16^INK4a^ protein in HUVECs expressing ectopic MEOX1 (MX1), MEOX2 (MX2), but not DNA binding mutant MEOX2^Q235E^ (Q235E). Total protein was isolated from HUVECs 72 hours after adenoviral transduction at 250 MOI. C) Quantification of the relative amount of p16^INK4a^ protein compared to EGFP transduced HUVECs. The intensity of the p16^INK4a^ band was normalized to the tubulin loading control. D) Schematic diagram of the human p16^INK4a^ promoter luciferase construct used in this paper. The base pair positions are indicated relative to the translational start site. E) Activation of the luciferase reporter gene from the 564 bp p16^INK4a^ promoter by wild type MEOX1 and MEOX2 but not by the DNA binding domain mutant versions of MEOX2 (MEOX2^Q235E^) or MEOX1 (MEOX1^Q220E^). Luciferase assays were performed in HUVECs. * Indicates a statistically significant change (p<0.05) when compared to the empty vector or EGFP control. ♦ Indicates a statistically significant difference (p<0.05) between MEOX1 and MEOX2.

p16^INK4a^ has also been shown to be a transcriptional target of MEOX2. Therefore, we wanted to compare the activation of p21^CIP1/WAF1^ and p16^INK4a^ by the two MEOX proteins in endothelial cells. First, we assessed the induction of endogenous p16^INK4a^ expression in HUVECs and found that MEOX1 is a more potent activator (three fold) of p16^INK4a^ gene expression as compared to MEOX2 (Figure 3A). Similar to what was observed for the mRNA expression, both MEOX1 and MEOX2 increased p16^INK4a^ protein levels, however the induction of p16^INK4a^ protein by MEOX1 was much greater (Figure 3B, 3C). Although we observed a small but significant increase in p16^INK4a^ mRNA expression with MEOX2^Q235E^, this activation did not result in increased protein expression, as assessed by western blot analysis (Figure 3A, 3B, 3C). Next, we used a 564 bp p16^INK4a^ promoter previously shown to be responsive to MEOX2 [16] in U2OS osteosarcoma cells. This p16^INK4a^ promoter construct contains two putative homeodomain binding sites (Figure 3D). In luciferase reporter gene assays, both MEOX1 and MEOX2 activated transcription from the 564 bp p16^INK4a^ promoter in HUVECs, when compared to the empty vector control; however, MEOX1 was a significantly more potent inducer of the p16^INK4a^ reporter gene than MEOX2 (Figure 3E). The DNA binding mutants, MEOX1^Q220E^ and MEOX2^Q235E^, did not activate transcription from this p16^INK4a^ promoter in HUVECs (Figure 3E). Taken together, this suggests that the ability of MEOX1 and MEOX2 to bind DNA, via the homeodomain, is required to activate p16^INK4a^ expression.

### MEOX1 and MEOX2 bind only the proximal homeodomain binding site within the p16^INK4a^ promoter

Subsequently, we wanted to determine which of the two putative homeodomain binding sites in the 564 bp p16^INK4a^ promoter were bound by MEOX1 and MEOX2. Since MEOX1^Q220E^ and MEOX2^Q235E^ did not activate p16^INK4a^ expression, we hypothesized that MEOX activation of p16^INK4a^ requires DNA binding and therefore the MEOX proteins would bind to one, or both, of the homeodomain binding sites within this promoter. To address this question, we designed EMSA probes which contained either the distal or proximal homeodomain binding sites.

**Figure 4:**
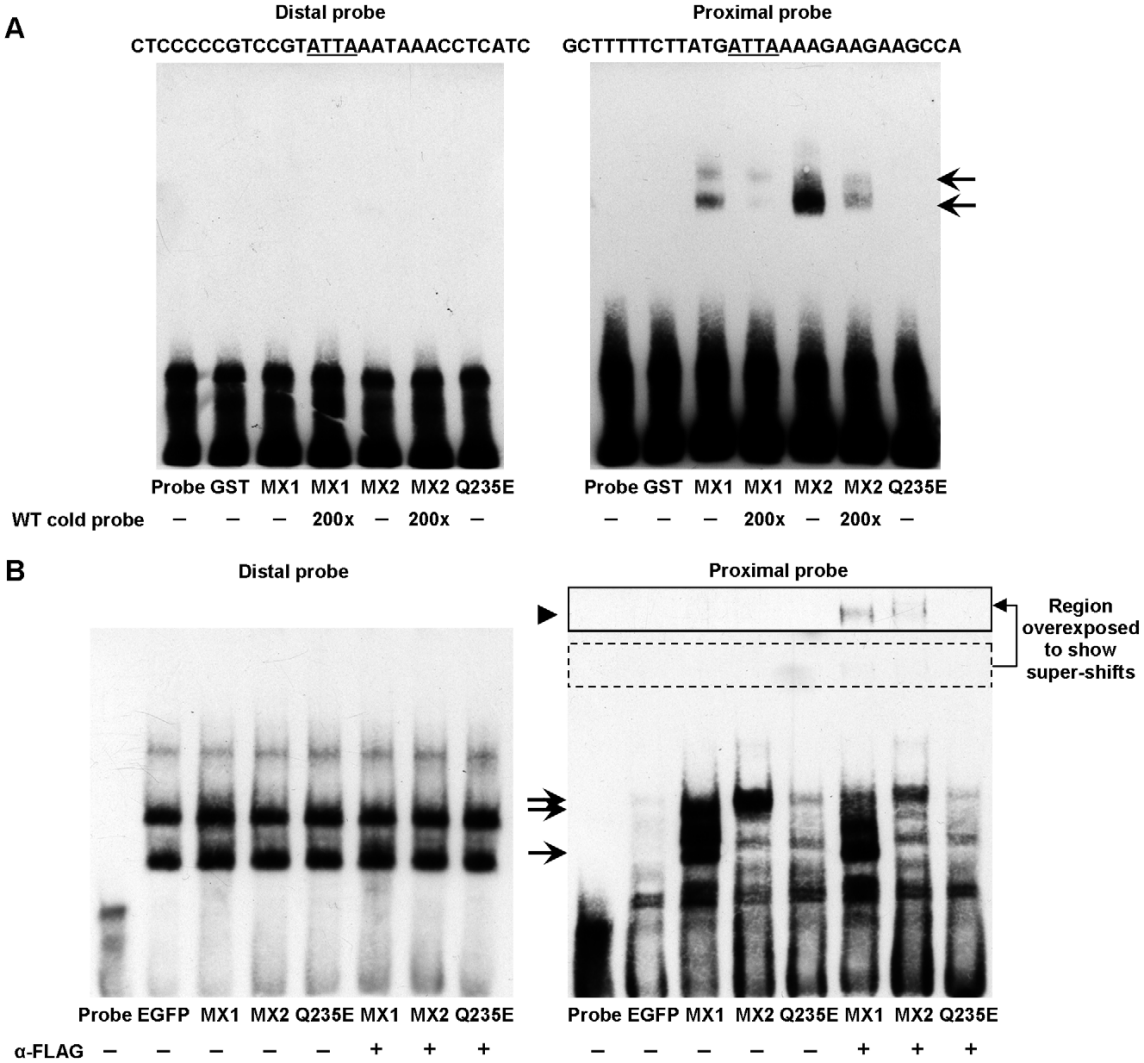
MEOX1 and MEOX2 bind to the proximal homeodomain binding site in the p16^INK4a^ promoter. Electrophoretic mobility shift assays were used to assess the ability of the MEOX proteins to bind to the homeodomain binding sites within the p16^INK4a^ luciferase promoter. The DNA probes each contained one homeodomain binding site and correspond to −833 to −862 bp (Distal) and −538 to −567 bp (Proximal) upstream of the p16^INK4a^ translation start site. A) Neither recombinant GST-tagged MEOX1 (MX1) nor MEOX2 (MX2) bound to the Distal probe (left). Both MEOX1 and MEOX2 clearly bound to the Proximal probe (right, arrow), while MEOX2^Q235E^ (Q235E) and GST did not. B) Nuclear extracts from HUVECs expressing N-terminally FLAG tagged MEOX1 or MEOX2 were not able to shift the Distal probe since no unique complexes were seen upon their expression (left). Incubation of nuclear extracts from HUVECs infected with MEOX1 or MEOX2 with the Proximal probe resulted in the formation of distinct complexes (arrows) (right), indicating that both MEOX proteins can bind to this sequence. Addition of FLAG antibody caused this protein-probe complex to super-shift (arrowhead), confirming that the observed shift is a MEOX protein-probe complex. Incubation of nuclear extracts from HUVECs expressing MEOX2^Q235E^ were unable to cause a specific shift of the DNA probes and a super-shift was not observed in the presence of FLAG antibody. Nuclear extracts from HUVECs expressing enhanced green fluorescent protein (EGFP) were used as a negative control.

With both recombinant proteins (Figure 4A) and HUVEC nuclear extracts (Figure 4B), we observed that MEOX1 and MEOX2 bound to a DNA probe which contains the proximal, but not the distal homeodomain binding site. As predicted, MEOX2^Q235E^ did not bind to either of the p16^INK4a^ probes (Figure 4A, 4B). When the Distal probe was incubated with recombinant GST-tagged MEOX proteins, no protein-DNA complexes were formed (Figure 4A, left). Incubation of the Distal probe with HUVEC nuclear extracts produced three different sized complexes; however, none of these complexes were specific to MEOX protein expression (Figure 4B, left). In contrast, incubation of the Proximal probe with nuclear lysates derived from either MEOX1 or MEOX2 transduced cells produced specific protein-DNA complex formation (Figure 4A, right; 4B, right). Intriguingly, two different sized complexes were observed in the presence of MEOX1, but only one complex was seen in the presence of MEOX2. To ensure that the shifts seen when the nuclear extracts from HUVECs expressing FLAG tagged MEOX1 or MEOX2 were due to MEOX protein/Proximal probe interaction, we added an anti-FLAG antibody to the reaction mixture. Binding of the anti-FLAG antibody to the specific MEOX protein/Proximal probe complexes caused these complexes to super-shift, resulting in the formation of a larger complex (Figure 4B, right, arrowhead; Figure S3, panel A) and a corresponding reduction in the intensity of the smaller complex (Figure 4B, right, arrows; Figure S3, panel A).

To confirm that MEOX protein/Proximal probe interaction was dependent upon the homeodomain binding site within the probe, the ATTA motif (WT) was mutated to AGGA (MT). Competition reactions show that the WT probe, but not the MT probe, competed for binding to MEOX1 and MEOX2 (Figure S3, panel B).

### Increased MEOX1 or MEOX2 expression leads to increased endothelial cell senescence

**Figure 5:**
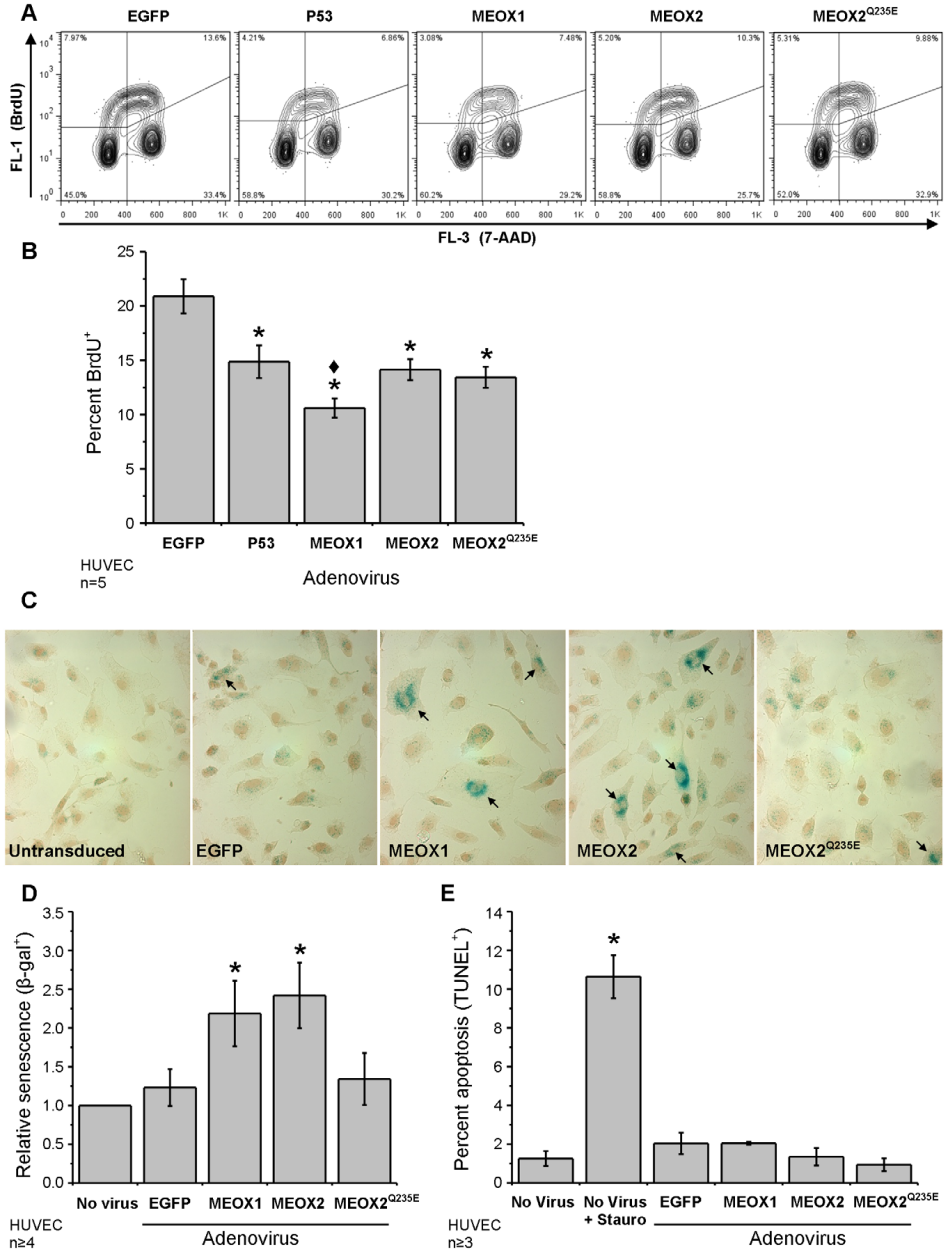
Increased MEOX1 or MEOX2 expression leads to increased endothelial cell senescence. A) Representative flow cytometry showing the density of BrdU^+^ endothelial cells (upper left and right quadrants). HUVECs were transduced with N-terminal FLAG tagged MEOX1 and MEOX2 adenoviral constructs at a multiplicity of infection (MOI) of 100; 48 hours later, cells were labeled with BrdU for one hour prior to fixation. DNA was stained with 7-aminoactinomycin D (7-AAD). B) Quantification of the flow cytometry data. Expression of MEOX1, MEOX2 and MEOX2^Q235E^ mutant decreased cellular proliferation comparable to p53 (positive control) as assessed by BrdU incorporation into cycling cells. C) Representative images showing SA-β-gal^+^ cells (blue). Nuclei were stained with hematoxylin (brown). D) Quantification of SA-β-gal^+^ cells shows that both MEOX1 and MEOX2 expression increased the number of senescent HUVECs. In contrast, MEOX2^Q235E^ expression did not alter the level of endothelial cell senescence. HUVECs were transduced with N-terminal FLAG tagged MEOX1 and MEOX2 adenoviral constructs at a MOI of 250; 48 hours later cells were fixed and stained. D) MEOX proteins do not induced endothelial cell apoptosis. HUVECs were transduced with FLAG tagged MEOX1, MEOX2 and MEOX2^Q235E^ adenoviral constructs at a MOI of 250; 48 hours later cells were fixed and stained. Staurosporine was used as a positive control for apoptosis induction. * Indicates a statistically significant change (p<0.05) when compared to the EGFP control. ♦ Indicates a statistically significant difference (p<0.05) between MEOX1 and MEOX2.

As p21^CIP1/WAF1^ and p16^INK4a^ are involved in the regulation of the cell-cycle, we tested whether MEOX1 and MEOX2 could induce changes in cellular proliferation. 5-bromo-2-deoxyuridine (BrdU) incorporation into the DNA of cycling HUVECs was used to detect changes in the percentage of cells in S phase of the cell cycle. As expected, expression of p53 in HUVECs (positive control) resulted in a significant decrease in the percentage of S phase cells as compared to the EGFP control (Figure 5A, 5B). Likewise, we observed a decrease in the proportion of S phase cells when MEOX1 or MEOX2 were expressed in HUVECs (Figure 5A, 5B). MEOX1 had the greatest effect on cellular proliferation and MEOX2^Q235E^ decreased the proportion of S phase cells to the same extent as wild-type MEOX2 (Figure 5A, 5B).

Unlike quiescence (reversible cell cycle arrest), senescence is a state of permanent cell cycle arrest and is associated with an increase in both p21^CIP1/WAF1^ and p16^INK4a^ protein expression. Thus, we ascertained whether the increased p21^CIP1/WAF1^ and p16^INK4a^ expression induced by MEOX1 and MEOX2 results in increased endothelial cell senescence. When MEOX1 or MEOX2 was expressed in HUVECs, we observed a significant increase in the number of senescent cells (approximately 2-fold) (Figure 5D), as assessed by senescence-associated β-galactosidase staining (Figure 5C). This corresponds to an increase from 4.5% senescence to 9.8% and 10.9% senescence, when comparing untransduced cells to cells ectopically expressing MEOX1 and MEOX2, respectively. Interestingly, expression of DNA binding mutant MEOX2^Q235E^ in HUVECs did not result in increased endothelial senescence (Figure 5C, 5D).

In addition to cell cycle arrest, p21^CIP1/WAF1^ regulates apoptosis. Changes in the percentage of apoptotic cells was measured by terminal deoxynucleotidyl transferase mediated dUTP nick end labeling (TUNEL). We did not observe increased apoptosis when MEOX1 or MEOX2 was expressed in HUVECs (Figure 5E). Furthermore, we did not observe any changes in necrotic cell death, as assessed by live/dead (calcein AM/ethidium homodimer-1) assay (data not shown). Thus, cell cycle inhibition by the MEOX proteins does not lead to increased endothelial cell death, under these conditions.

### MEOX1 and MEOX2 activate transcription from a minimal p21^CIP1/WAF1^ promoter independent of its ability to bind DNA

**Figure 6:**
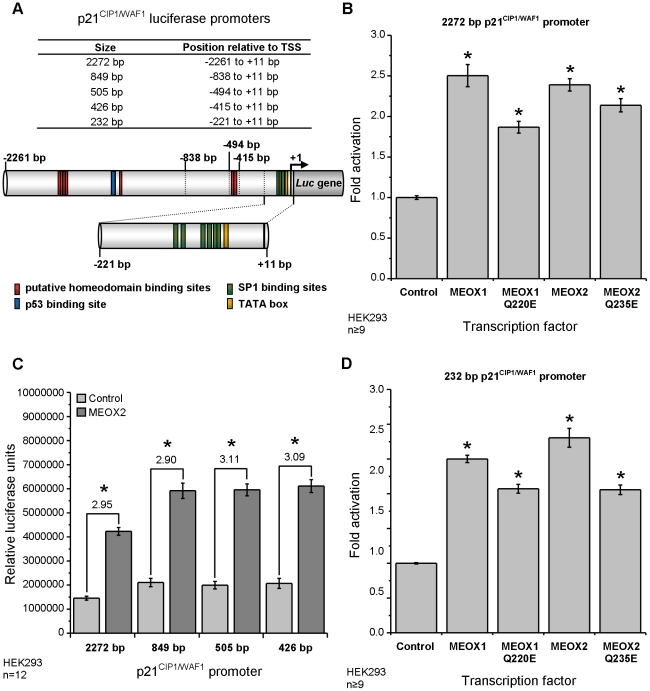
MEOX2 activates transcription from a minimal p21^CIP1/WAF1^ promoter independent of its ability to bind DNA. A) Schematic diagram of the human p21^CIP1/WAF1^ promoter luciferase constructs used in this paper. The 5′ termini are indicated relative to the transcriptional start site (arrow). Relevant transcription factor binding sites (p53, homeodomain, SP1 and TATA) are also shown. B) Activation of the luciferase reporter gene from the 2272 bp p21^CIP1/WAF1^ promoter by wild type MEOX1, MEOX2 and their respective DNA binding mutant versions MEOX1^Q220E^ and MEOX2^Q235E^. C) Comparison of MEOX2 activation from the 2272 bp, 849 bp, 505 bp and 426 bp p21^CIP1/WAF1^ promoters. The fold activation by MEOX2 compared to the empty vector control is indicated for each promoter. D) Activation of the luciferase reporter gene from the minimal 232 bp p21^CIP1/WAF1^ promoter by wild type MEOX1, MEOX2 and their respective DNA binding mutant versions MEOX1^Q220E^ and MEOX2^Q235E^. B-D). All luciferase assays were performed in HEK293 cells. * Indicates a statistically significant change (p<0.05), when compared to the empty vector control.

We first established the level of MEOX1 and MEOX2 induced transcription of a luciferase reporter gene from a 2272 bp human p21^WAF1/CIP1^ promoter [33] in HEK293 cells. This 2272 bp p21^CIP1/WAF1^ promoter has been previously shown to be activated by MEOX2 [25]. It contains several transcription factor binding sites, including one p53 binding site, seven putative homeodomain binding sites and six SP1 binding sites (Figure 6A). MEOX1 and MEOX2 induced greater than two fold activation of the luciferase reporter from the 2272 bp p21^CIP1/WAF1^ promoter, when compared to the empty vector control (Figure 6B). Interestingly, the DNA binding deficient MEOX1^Q220E^ and MEOX2^Q235E^ proteins were able to activate this luciferase reporter to a level comparable to wild-type proteins (Figure 6B).

Progressive truncation of the 2272 bp p21^CIP1/WAF1^ promoter was performed in order to determine the minimal promoter that is sufficient for MEOX2 induced transcription activation. It was previously shown that MEOX2 activation of the p21^CIP1/WAF1^ promoter is independent of p53 activity [25]. Indeed, MEOX2 equivalently activated an 849 bp p21^CIP1/WAF1^ promoter, which lacks the p53 binding site (Figure 6C). Furthermore, deletion of the putative homeodomain binding sites did not affect MEOX2 induced transcription from the p21^CIP1/WAF1^ promoter, as demonstrated by the ability of MEOX2 to activate a 426 bp p21^CIP1/WAF1^ promoter (Figure 6C). Significant activation of the luciferase reporter by MEOX1 and MEOX2 was still seen with the smallest promoter tested, which contained only the most proximal 232 bp of the p21^CIP1/WAF1^ promoter (Figure 6D). This promoter contains multiple SP1 binding sites, but no homeodomain binding sites. MEOX1^Q220E^ and MEOX2^Q235E^ were also both able to activate transcription from the 232 bp p21^CIP1/WAF1^ promoter (Figure 6D), suggesting that MEOX1 and MEOX2 do not need to bind DNA in order to induce p21^CIP1/WAF1^ transcription. Notably, MEOX2^Q235E^ was able to activate endogenous p21^CIP1/WAF1^ expression at the mRNA and protein levels to equivalent degrees as observed for wild-type MEOX2 (Figure 2A, 2B, 2C). This result further demonstrated that activation of the p21^CIP1/WAF1^ gene by MEOX2 is DNA-binding independent.

In order to verify that MEOX1^Q220E^ and MEOX2^Q235E^ are unable to bind to DNA, we performed electrophoretic mobility shift assays (EMSAs) using a sequence from the p21^CIP1/WAF1^ promoter previously shown to contain a MEOX2 binding site [15] (Figure S4; Figure S5, panel D). This sequence is not contained within the 2272 bp p21^CIP1/WAF1^ luciferase promoter; it is located approximately 9.5 kb upstream of the p21^CIP1/WAF1^ transcription start site. As demonstrated using recombinant GST tagged proteins as well as nuclear extracts from HUVECs and HEK293 cells expressing FLAG tagged MEOX proteins (Figure S4, panels A,C; Figure S5, panel D), both MEOX1 and MEOX2 were able to bind this probe. Unlike wild-type MEOX proteins, no shift was observed with MEOX1^Q220E^ (Figure S5, panel D) or MEOX2^Q235E^ (Figure S4, panels A,C; Figure S5, panel D), indicating that these proteins are indeed unable to bind to this probe. GST alone (Figure S4, panels A,B), nuclear extracts from EGFP expressing HUVECs (Figure S4, panels C,D) and nuclear extracts from HEK293 cells transfected with empty vector (Figure S5, panel D) were used as negative controls for DNA binding.

### Role of SP1 in mediating effects of MEOX1 and MEOX2 on p21^CIP1/WAF1^ transcription

**Figure 7:**
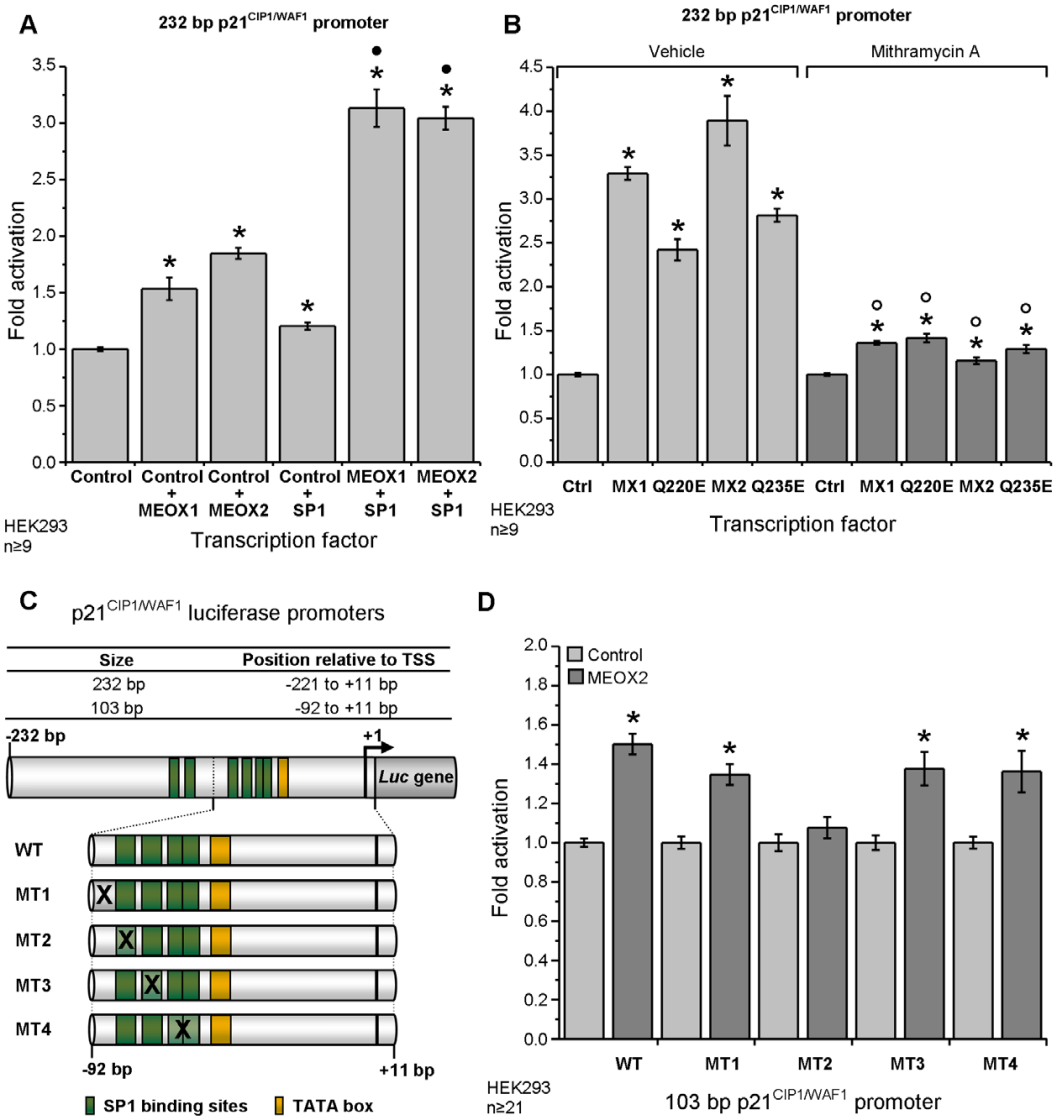
MEOX1 and MEOX2 activation of the p21^CIP1/WAF1^ promoter is dependent on SP1 function. A) Co-transfection of SP1 enhanced the activation of the 232 bp p21^CIP1/WAF1^ promoter by both MEOX1 and MEOX2. B) Treatment of cells with mithramyin A but not vehicle (methanol) for 24 hours blocked MEOX1 and MEOX2 mediated activation of the reporter gene from the 232 bp p21^CIP1/WAF1^ promoter. C) Schematic diagram of the human 103 bp p21^CIP1/WAF1^ promoter luciferase constructs used in this paper. D) Activation of the luciferase reporter gene from the 103 bp p21^CIP1/WAF1^ promoter by MEOX2 was abolished through mutation of the most upstream SP1 site. WT  =  Wild-type promoter, MT  =  mutant promoters. * Indicates a statistically significant change (p<0.05), when compared to the empty vector control. • Indicates a statistically significant difference (p<0.05) between MEOX+Control and MEOX+SP1. ○ Indicates a statistically significant difference (p<0.05) between vehicle and mithramycin A treatment.

Our data identifies the MEOX responsive region of the p21^WAF1/CIP1^ promoter as residing within the most proximal 232 bp, through which it activates transcription independent of DNA binding. This region does not contain homeodomain binding sites but does contain several SP1 binding sites. To interrogate the role of SP1 in mediating MEOX activation of the p21^CIP1/WAF1^ promoter, we co-transfected MEOX1 or MEOX2 with and without SP1. We determined that either MEOX1 or MEOX2, together with SP1, synergistically activated transcription from the 232 bp p21^CIP1/WAF1^ promoter (Figure 7A). To inhibit SP1 binding, we treated cells that were transfected with the 232 p21^CIP1/WAF1^ promoter and MEOX expression plasmids with mithramycin A. Mithramycin A binds GC-rich regions of DNA and thereby inhibits SP1 interaction with its binding sites [37], [38]. This treatment blocked the MEOX mediated activation of the 232 bp p21^CIP1/WAF1^ promoter (Figure 7B). Next, we studied the ability of MEOX2 to activate transcription from a 103 bp p21^CIP1/WAF1^ promoter with wild-type or mutated SP1 binding sites (Figure 7C) [34], [35]. Only mutation of the most upstream SP1 binding site abolished the ability of MEOX2 to activate transcription from this promoter (Figure 7D).

## Discussion

Our results demonstrate for the first time that MEOX1 regulates the MEOX2 target genes p21^CIP1/WAF1^ and p16^INK4a^. We observed that increased expression of the MEOX homeodomain transcription factors leads to both cell cycle arrest and endothelial cell senescence. Furthermore we showed that the mechanism of transcriptional activation of these cyclin dependent kinase inhibitor genes by MEOX1 and MEOX2 is distinct; the MEOX proteins activate p16^INK4a^ in a DNA-binding dependent manner, whereas they induces p21^CIP1/WAF1^ in a DNA binding independent manner, which requires SP1.

Knockout of both MEOX1 and MEOX2 in mice produces a phenotype that is more severe than what the additive phenotypes of the single MEOX gene knockout mice would be [30], indicating that these proteins have partially redundant functions. Our data provides evidence that the MEOX transcription factors regulate the expression of similar sets of target genes. In support of this notion, the amino acid composition of the MEOX1 and MEOX2 homeodomains (HD) is nearly identical (Figure 1A). Indeed, MEOX2 has been shown by EMSA to bind to the MEOX1 binding site within the NKX3-2/BapX1 promoter [31]. Additionally, we show that MEOX1 and MEOX2 are able to bind DNA probes which contain homeodomain binding sites from the p21^CIP1/WAF1^ and p16^INK4a^ promoters (Figure 4, Figure S4).

Aside from the homeodomain, there is only limited sequence conservation between MEOX1 and MEOX2. Differences in MEOX1 versus MEOX2 activation of common target genes are likely to be due to: i) the presence of unique transactivation motifs within these proteins, or ii) the ability of these proteins to interact with distinct transcriptional co-factors via protein-protein interaction domains. The HQ domain of MEOX2 was shown to be important for the ability of MEOX2 to activate transcription from a 2.4 Kb p21^CIP1/WAF1^ promoter, as deletion of this domain dramatically decreased MEOX2 induced reporter gene expression [15]. Similar polyhistidine/polyglutamine rich motifs are found in other human proteins [43], [44], such as the homeodomain transcription factor HOXA1, a known transcriptional activator [45]. Interestingly, MEOX1 does not contain an HQ rich domain, but is still able to activate transcription of p21^CIP1/WAF1^ and p16^INK4a^. Surprisingly, deletion of the MEOX2 HQ rich domain did not alter protein localization, expression or function (Figure S5).

Unlike the HQ domain, there are currently no known functions for the MID domain. Outside of the homeodomain, the MID domain is the next most highly conserved domain between MEOX1 and MEOX2 (Figure 1A). Furthermore, the MID region, along with the homeodomain, was shown to be important in the regulation of the p16^INK4a^ gene [16]. In addition, this region of MEOX2 is sufficient for binding to vascular endothelial zinc-finger 1 (VEZF1) protein in yeast-two-hybrid assays (unpublished observation). These results suggest that the MID domain of the MEOX proteins may play a role in mediating protein-protein interactions with other transcription factors.

MEOX1 and MEOX2 differ slightly in subcellular localization; both MEOX1 and MEOX2 were detected throughout the nucleus, however MEOX1 was also detected in the cytosol (Figure 1C, 1D). Although the reason for this difference in localization is not known, we speculate that unique protein binding partners may explain the cytosolic localization of MEOX1. Despite the difference in cytosolic protein, both MEOX1 and MEOX2 are transcriptionally active within the nucleus, as shown by their ability to increase the expression of the p21^CIP1/WAF1^ and p16^INK4a^ genes.

Although p16^INK4a^ is a known target of MEOX2 regulation, to our surprise MEOX1 was a much stronger activator of p16^INK4a^ expression in HUVECs (Figure 3A, 3B, 3C). In contrast, MEOX1 was a weaker inducer of p21^CIP1/WAF1^ protein expression, when compared to MEOX2 (Figure 2C). We observed that MEOX1 had the greatest effect on inhibiting cellular proliferation (Figure 5A, 5B), conceivably due to its strong induction of p16^INK4a^ expression. However, despite the difference in p21^CIP1/WAF1^ and p16^INKa^ expression, MEOX1 and MEOX2, were similarly able to induce senescence (Figure 5B, 5C), further supporting the hypothesis that the co-ordinated up-regulation of both p21^CIP1/WAF1^ and p16^INK4a^ are required for the induction of the cellular senescence program in HUVECs. To our knowledge, the MEOX proteins are the second instance of homeodomain transcription factors capable of inducing senescence. Ectopic expression of VentX, a transactivator of p16^INK4a^, has recently been shown to cause senescence in cancer cells [46]. Corresponding to our results, these authors show that both p21^CIP1/WAF1^ and p16^INK4a^ are involved in permanent cell cycle arrest.

In contrast to p21^CIP1/WAF1^, we show that MEOX1 and MEOX2 require the ability to bind to DNA in order to activate transcription of the p16^INK4a^ gene. Unlike wild-type MEOX1 and MEOX2, MEOX1^Q220E^ and MEOX2^Q235E^ were unable to induce p16^INK4a^ expression in HUVECs (Figure 3B, 3C, 3E). Furthermore, ectopic MEOX2^Q235E^ expression decreased the proportion of S phase cells to the same extent as wild-type MEOX2 (Figure 5B), but did not lead to increased senescence in HUVECs (Figure 5D). This finding suggests that p21^CIP1/WAF1^ is the main inhibitor of cell cycle progression in response to MEOX2 expression; however both p21^CIP1/WAF1^ and p16^INK4a^ are required for the induction of the cellular senescence program in HUVECs. Alternatively, MEOX2 may activate other genes required for senescence in a DNA binding dependent manner.

Canonically, control of target gene transcription by homeodomain proteins is achieved through direct binding of DNA via the homeodomain. However, the homeodomain has also been shown to act as a critical protein-protein interaction domain in several homeodomain proteins, including MEOX2 [47], [48], thereby permitting homeodomain transcription factors to modify target gene transcription without binding DNA directly. For our study, we created DNA binding deficient versions of the MEOX1 and MEOX2 proteins (MEOX1^Q220E^ and MEOX2^Q235E^) by mutating the amino acid at position 50 of the homeodomain from a positively charged glutamine to a negatively charged glutamate (Figure 1A). Mutation of this highly conserved residue within the homeodomain has been used previously to effectively abolish DNA binding of other homeodomain transcription factors [40], [41]. MEOX1^Q220E^ and MEOX2^Q235E^ are full-length proteins and are localized to the nucleus like wild-type MEOX proteins (Figure 1C, 1D). Therefore, mutation of the MEOX homeodomain abolished DNA binding (Figure 4A, 4B; Figure S4, panels A,C; Figure S5, panel D), but is unlikely to affect the ability of MEOX proteins to form protein-protein interactions. In contrast, the homeodomain deleted version of MEOX2 (MEOX2^K195_K245del^) was localized to punctate nuclear aggregates and was consistently detected in the cytoplasm (Figure 1C, 1D). The cytoplasmic localization of the MEOX2^K195_K245del^ protein occurred despite the predicted nuclear localization signal being left intact. The punctate nuclear aggregates of homeodomain deleted MEOX2 have been shown to co-localize with splicing factor SC35, a component of the nuclear speckles [44]. Thus, the deletion of the MEOX2 homeodomain resulted in a loss of DNA binding but also likely affected protein function due to altered localization and disrupted protein-protein interactions.

MEOX2 activation of the p21^CIP1/WAF1^ gene was reported to be dependent upon DNA binding, as a homeodomain deleted version of MEOX2 could not activate transcription from a 2.4 Kb p21^CIP1/WAF1^ promoter [15], [25]. However, we postulate that the homeodomain deleted MEOX2 is unable to activate transcription from the p21 promoter because of its improper subcellular localization which may make it unable to associate with either the p21 gene or requisite transcriptional co-factors.

In this study we show that MEOX2^Q235E^ was as effective as wild-type MEOX2 at inducing p21^CIP1/WAF1^ expression in primary endothelial cells (Figure 2A, 2B, 2C). Furthermore, removal of all the putative homeodomain binding sites from the p21^CIP1/WAF1^ promoter did not affect the ability of MEOX1 or MEOX2 to activate transcription from the p21^CIP1/WAF1^ promoter (Figure 6D). Combined, this led us to conclude that MEOX activation of the p21^CIP1/WAF1^ gene is DNA-binding independent, and likely occurs through interaction with other transcription co-factors. MEOX1, MEOX1^Q220E^, MEOX2 and MEOX2^Q235E^ were all able to activate transcription from the 232 bp p21^CIP1/WAF1^ luciferase promoter which contains six SP1 binding sites (Figure 6A, 6D). Thus, we hypothesized that the MEOX proteins may interact with SP1 or co-operate with other transcription factors which activate the p21^CIP1/WAF1^ gene from this minimal region [49]–[58]. Indeed, increasing SP1 expression enhanced MEOX mediated activation, whereas mithramycin A blunted MEOX mediated activation, of the 232 bp p21^CIP1/WAF1^ luciferase promoter (Figure 7A, 7B). Furthermore, we show that mutation of a single SP1 site in a 103 bp p21^CIP1/WAF1^ luciferase promoter abolished MEOX2 induced activation (Figure 7D).

Age is a major risk factor for cardiovascular diseases, such as atherosclerosis. As blood vessels age, they accumulate increasing numbers of senescent cells, leading to endothelial dysfunction and ultimately vascular disease [6], [7]. Indeed, human atherosclerotic tissue has been shown to contain a higher proportion of senescent cells [8]–[10]. Furthermore, children affected by Hutchison-Gilford Progeria Syndrome, a disease of premature aging, often die from accelerated atherosclerosis [7], [59]. Microarray analysis has shown that MEOX2 expression is increased 10-fold in cells from Hutchison-Gilford Progeria Syndrome patients [59]. Thus, MEOX proteins may play a role in endothelial aging and atherosclerosis, by inhibiting cell cycle progression and inducing endothelial cell senescence.

In summary, we show that MEOX1 can activate the known MEOX2 target genes p21^CIP1/WAF1^ and p16^INK4a^ in primary endothelial cells and induce endothelial senescence. In addition, our findings demonstrate that MEOX2 activates transcription of p21^CIP1/WAF1^ independent of DNA binding. Thus, future studies to identify novel MEOX1 and MEOX2 target genes will aim to determine whether activation occurs via DNA binding dependent or independent mechanisms. In addition, for DNA binding independent target genes, such as p21^CIP1/WAF1^, experiments will be done to identify transcription co-factors of the MEOX proteins. Together, these findings may provide further insight into how the MEOX proteins induce senescence and shed light on the role of the MEOX proteins in the pathogenesis of atherosclerosis.

## Supporting Information

Figure S1 **Ectopic MEOX proteins are expressed at similar levels.** N-terminally tagged MEOX proteins were detected using an anti-FLAG antibody and α-tubulin was used as a loading control. A) A representative western blot displaying the relative level of MEOX protein expression in HEK293 cells 24 hours after transfection. Each lane represents an independent transfection. B) Representative western blot displaying the relative level of MEOX protein expression in HUVECs 48 hours after adenoviral transduction at an MOI of 250. Each lane represents an independent transduction.
(TIF)

Figure S2 **The position or inclusion of the FLAG epitope does not affect MEOX2 function.** Luciferase assay demonstrating the ability of N-terminal, C-terminal and non-FLAG tagged MEOX2 proteins to activate a 2272 bp p21^CIP1/WAF1^ promoter. * Indicates a statistically significant change (p<0.05) when compared to the empty vector controls. n.s. denotes no statistically significant difference (p<0.05) in promoter activation is observed between the various MEOX2 proteins.
(TIF)

Figure S3 **MEOX proteins bind to the proximal homeodomain binding site in the p16^INK4a^ promoter.** A) Overexposure of the p16^INK4a^ EMSA shown in Figure 6B, left. Incubation of nuclear extracts from HUVECs infected with MEOX1 or MEOX2 with the Proximal probe resulted in the formation of distinct complexes (arrowhead) (left), indicating that both MEOX proteins can bind to this sequence. Addition of FLAG antibody caused this protein-probe complex to super-shift (arrowhead) (right), confirming that the observed shift is a MEOX protein-probe complex. Incubation of nuclear extracts from HUVECs expressing MEOX2^Q235E^ were unable to cause a specific shift of the DNA probes and a super-shift was not observed in the presence of FLAG antibody. Nuclear extracts from HUVECs expressing enhanced green fluorescent protein (EGFP) were used as a negative control. B) Binding of MEOX1 (right) and MEOX2 (left) to the Proximal probe could be competed with excess wild type (WT), but not mutant (MT) cold probe, in which the homeodomain binding site was abolished.
(TIF)

Figure S4 **MEOX1 and MEOX2, but not MEOX2^Q235E^, bind to a region of the p21^CIP1/WAF1^ promoter.** Electrophoretic mobility shift assays (EMSAs) were used to assess the DNA binding capabilities of the various MEOX proteins. The DNA probe contained two MEOX2 binding sites originating from the sequence −9519 bp to −9489 bp upstream of the p21^CIP1/WAF1^ transcription start site. A) Recombinant GST-tagged MEOX1 (MX1) and MEOX2 (MX2) bound to the probe (arrow) whereas the DNA binding domain mutant version of MEOX2 (Q235E) and GST alone did not. B) Binding of MEOX2 to the DNA probe could be competed with excess wild type (WT), but not with excess mutant (MT) cold probe, in which the homeodomain binding sites were mutated. C) Nuclear extracts from HUVECs expressing N-terminally FLAG tagged MEOX1 and MEOX2 resulted in distinct shifted complexes (arrows), that were not seen with the EGFP or MEOX2^Q235E^ nuclear extracts. Addition of FLAG antibody to nuclear extracts from MEOX1 and MEOX2 infected cells, but not EGFP or MEOX2^Q235E^ infected cells, resulted in the formation of a super-shift complex (arrowhead). D) Binding of the DNA probe by MEOX2 in endothelial cell nuclear extracts was competed with excess wild type (WT), but not mutant (MT) cold probe. Addition of FLAG antibody, but not non-immune IgG, caused the formation of a super-shift complex (arrowhead).
(TIF)

Figure S5 **Deletion of the HQ rich domain of MEOX2 does not alter protein expression, localization or function.** A) Schematic representation of the MEOX2^H68_Q85del^ protein compared to wild-type MEOX1 and MEOX2. B) A representative western blot demonstrating the subcellular localization of MEOX2^ H68_Q85del^ protein compared to wild-type MEOX2 in HEK293 cells, 48 hours after transfection. α-tubulin was used as a cytoplasmic (C) marker and lamin A/C was used as nuclear (N) marker. C) Representative fluorescent immunocytochemistry showing the localization and level of expression of the MEOX proteins in HEK293 cells 24 hours after transfection. The N-terminally tagged MEOX proteins were detected using an anti-FLAG antibody (green) and nuclei were stained with DAPI (blue). Empty vector was used as a negative control. D) Incubation of nuclear extracts from HEK293 cells transfected with MEOX1, MEOX2 or MEOX2^H68_Q85del^ with the p21 probe resulted in the formation of distinct complexes (arrows). Homeodomain mutated MEOX1^Q220E^ and MEOX2^Q235E^ were unable to cause a specific shift of the DNA probe. E) MEOX2^H68_Q85del^ activation of the luciferase reporter gene from the 232 bp p21^CIP1/WAF1^ promoter is comparable to wild type MEOX2.
(TIF)

Table S1 **List of PCR primers used to create MEOX1 and MEOX2 fusion proteins.**
(DOC)

Table S2 **List of PCR primers used to create the p21^CIP1/WAF1^ promoter luciferase constructs.**
(DOC)

Table S3 **List of PCR primers used for qRT-PCR.**
(DOC)

Table S4 **List of EMSA probes.**
(DOC)

Methods S1 **Supplementary methods.**
(DOC)
