# Supplementary figures and images for: Mechanisms of MEOX1 and MEOX2 Regulation of the Cyclin Dependent Kinase Inhibitors p21CIP1/WAF1 and p16INK4a in Vascular Endothelial Cells
Source: PLoS One. 2011 Dec 20;6(12):e29099. doi: 10.1371/journal.pone.0029099 (PMC3243699; doi:10.1371/journal.pone.0029099)

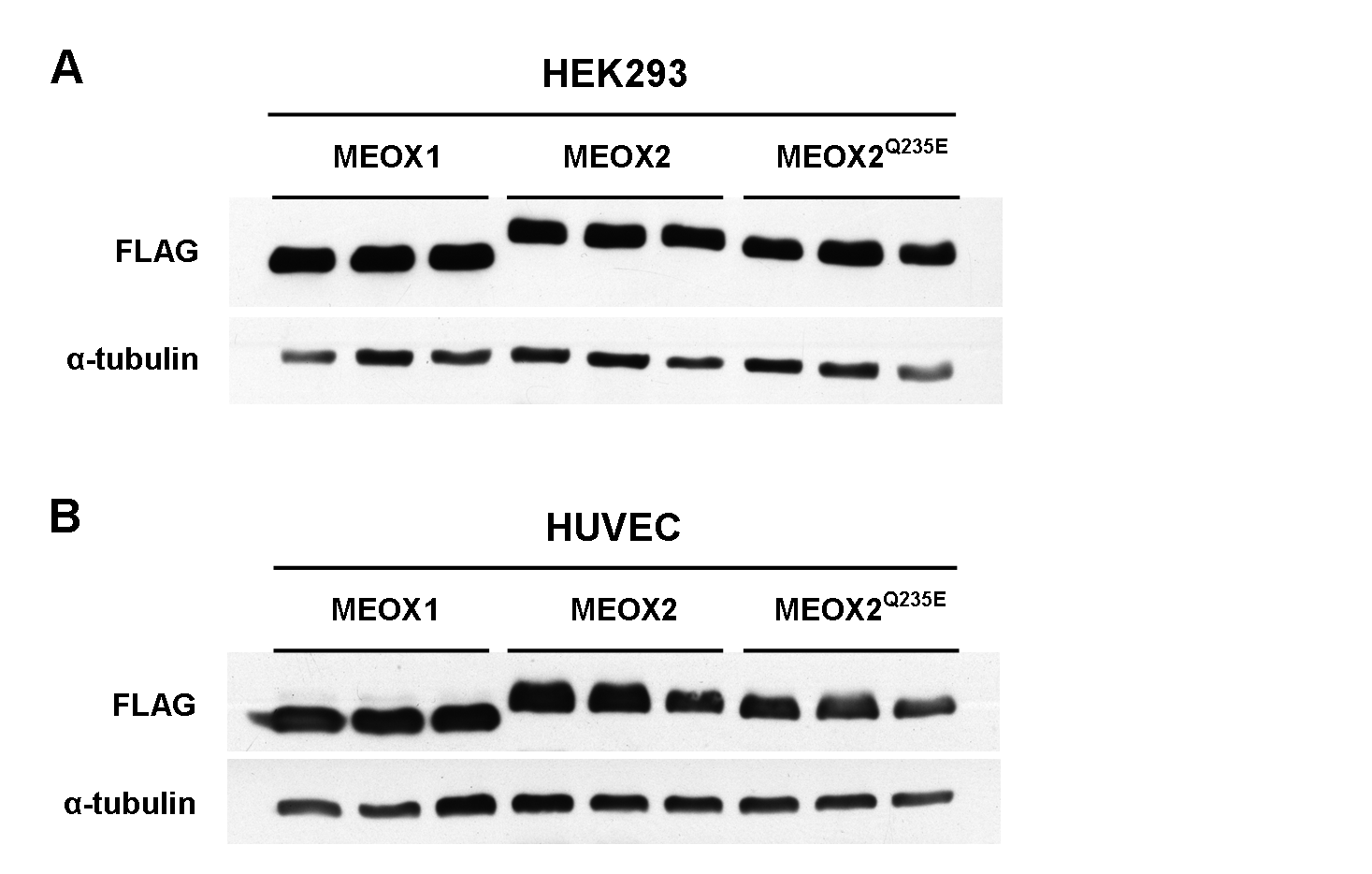

Supplement: Figure S1 — Ectopic MEOX proteins are expressed at similar levels. N-terminally tagged MEOX proteins were detected using an anti-FLAG antibody and α-tubulin was used as a loading control. A) A representative western blot displaying the relative level of MEOX protein expression in HEK293 cells 24 hours after transfection. Each lane represents an independent transfection. B) Representative western blot displaying the relative level of MEOX protein expression in HUVECs 48 hours after adenoviral transduction at an MOI of 250. Each lane represents an independent transduction. (TIF) [file pone.0029099.s001.tif]

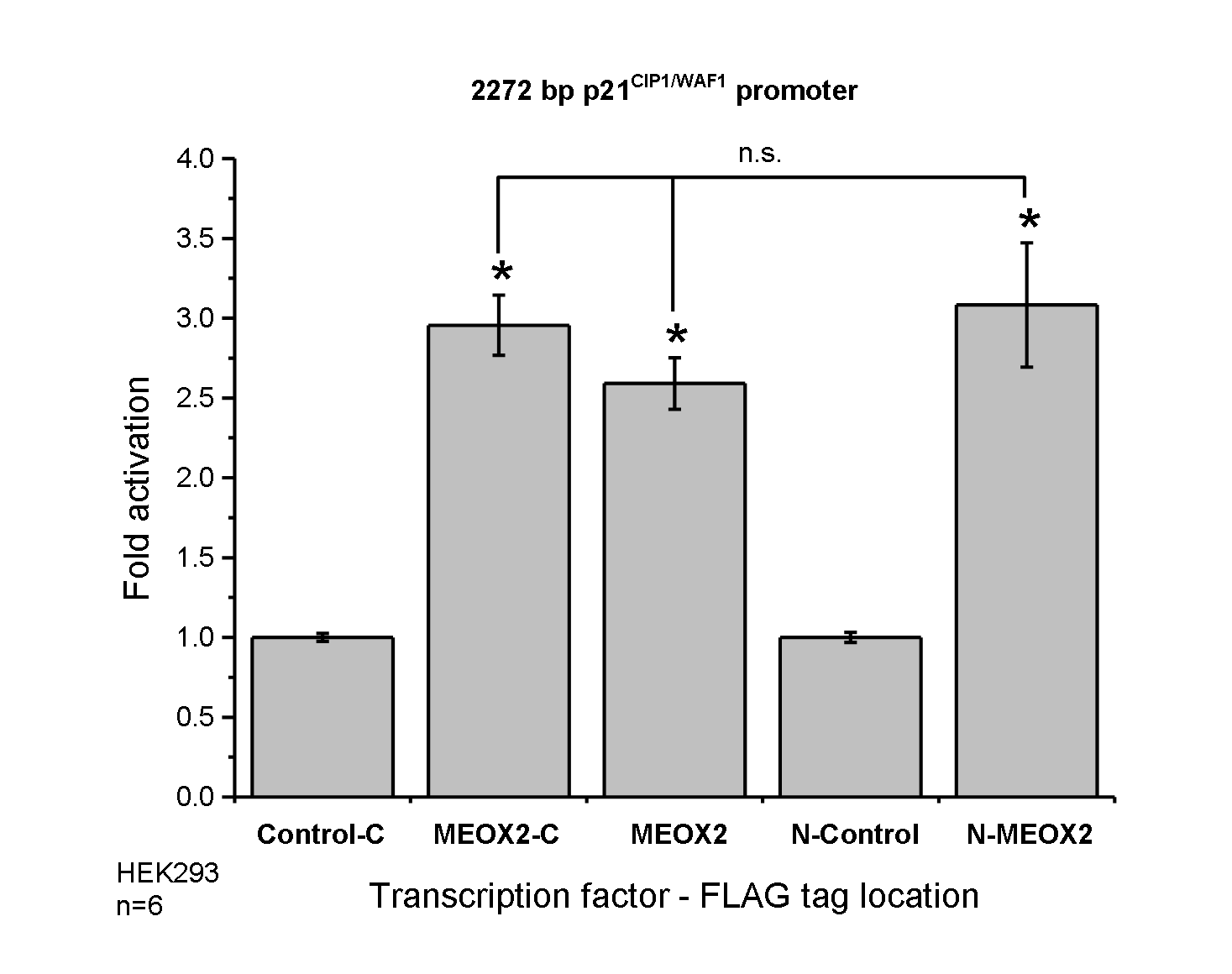

Supplement: Figure S2 — The position or inclusion of the FLAG epitope does not affect MEOX2 function. Luciferase assay demonstrating the ability of N-terminal, C-terminal and non-FLAG tagged MEOX2 proteins to activate a 2272 bp p21CIP1/WAF1 promoter. * Indicates a statistically significant change (p<0.05) when compared to the empty vector controls. n.s. denotes no statistically significant difference (p<0.05) in promoter activation is observed between the various MEOX2 proteins. (TIF) [file pone.0029099.s002.tif]

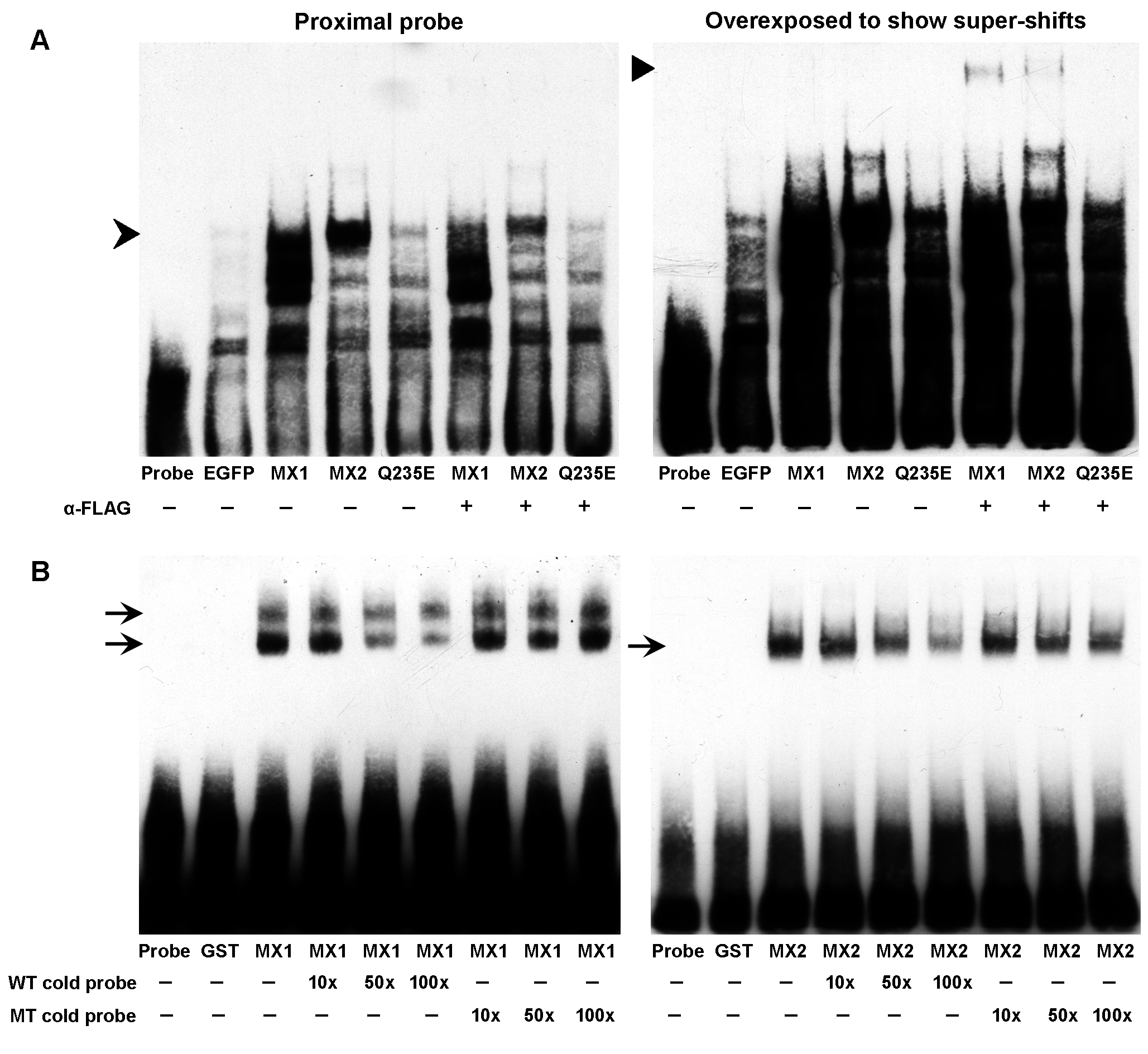

Supplement: Figure S3 — MEOX proteins bind to the proximal homeodomain binding site in the p16INK4a promoter. A) Overexposure of the p16INK4a EMSA shown in Figure 6B, left. Incubation of nuclear extracts from HUVECs infected with MEOX1 or MEOX2 with the Proximal probe resulted in the formation of distinct complexes (arrowhead) (left), indicating that both MEOX proteins can bind to this sequence. Addition of FLAG antibody caused this protein-probe complex to super-shift (arrowhead) (right), confirming that the observed shift is a MEOX protein-probe complex. Incubation of nuclear extracts from HUVECs expressing MEOX2Q235E were unable to cause a specific shift of the DNA probes and a super-shift was not observed in the presence of FLAG antibody. Nuclear extracts from HUVECs expressing enhanced green fluorescent protein (EGFP) were used as a negative control. B) Binding of MEOX1 (right) and MEOX2 (left) to the Proximal probe could be competed with excess wild type (WT), but not mutant (MT) cold probe, in which the homeodomain binding site was abolished. (TIF) [file pone.0029099.s003.tif]

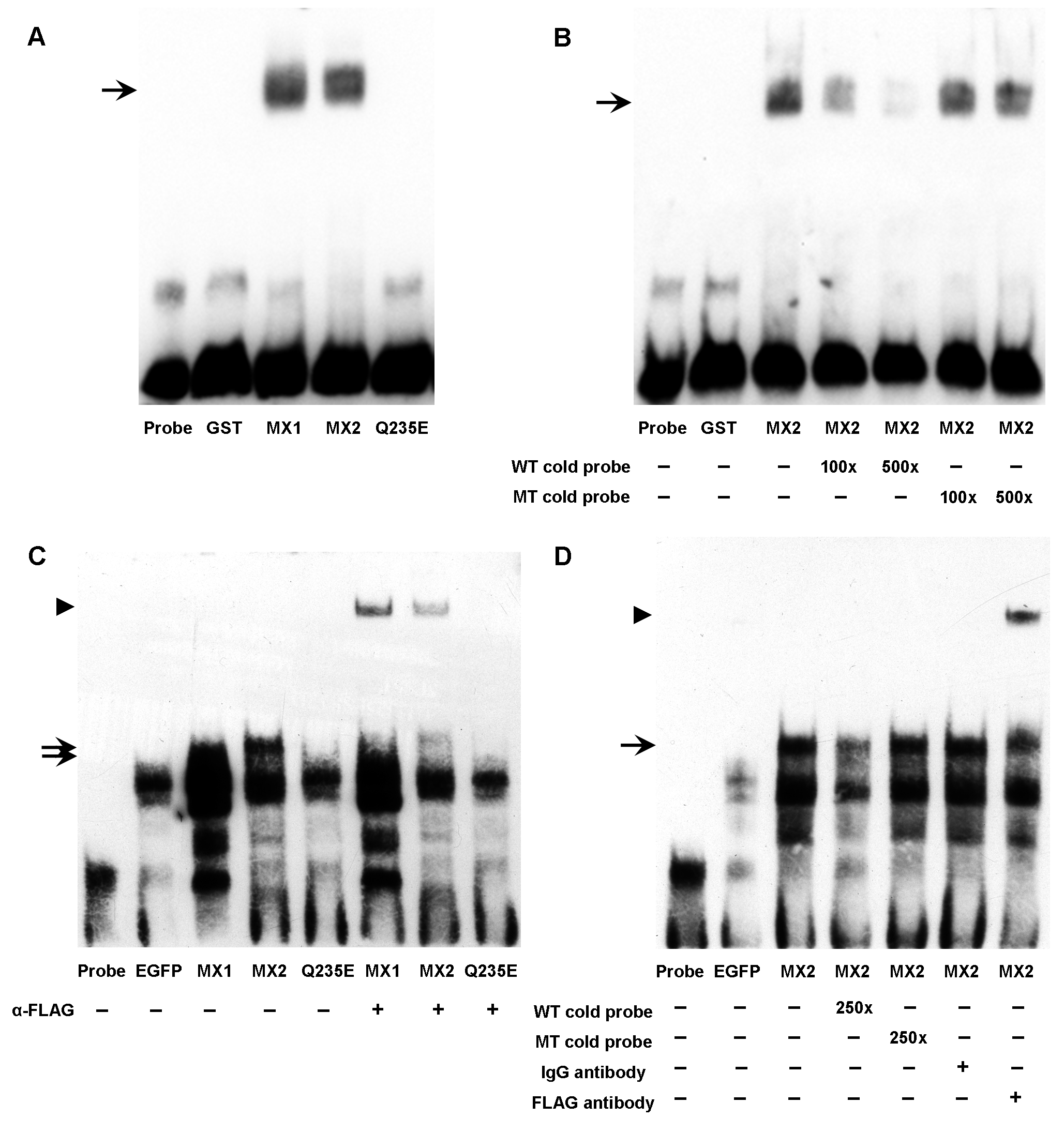

Supplement: Figure S4 — MEOX1 and MEOX2, but not MEOX2Q235E, bind to a region of the p21CIP1/WAF1 promoter. Electrophoretic mobility shift assays (EMSAs) were used to assess the DNA binding capabilities of the various MEOX proteins. The DNA probe contained two MEOX2 binding sites originating from the sequence −9519 bp to −9489 bp upstream of the p21CIP1/WAF1 transcription start site. A) Recombinant GST-tagged MEOX1 (MX1) and MEOX2 (MX2) bound to the probe (arrow) whereas the DNA binding domain mutant version of MEOX2 (Q235E) and GST alone did not. B) Binding of MEOX2 to the DNA probe could be competed with excess wild type (WT), but not with excess mutant (MT) cold probe, in which the homeodomain binding sites were mutated. C) Nuclear extracts from HUVECs expressing N-terminally FLAG tagged MEOX1 and MEOX2 resulted in distinct shifted complexes (arrows), that were not seen with the EGFP or MEOX2Q235E nuclear extracts. Addition of FLAG antibody to nuclear extracts from MEOX1 and MEOX2 infected cells, but not EGFP or MEOX2Q235E infected cells, resulted in the formation of a super-shift complex (arrowhead). D) Binding of the DNA probe by MEOX2 in endothelial cell nuclear extracts was competed with excess wild type (WT), but not mutant (MT) cold probe. Addition of FLAG antibody, but not non-immune IgG, caused the formation of a super-shift complex (arrowhead). (TIF) [file pone.0029099.s004.tif]

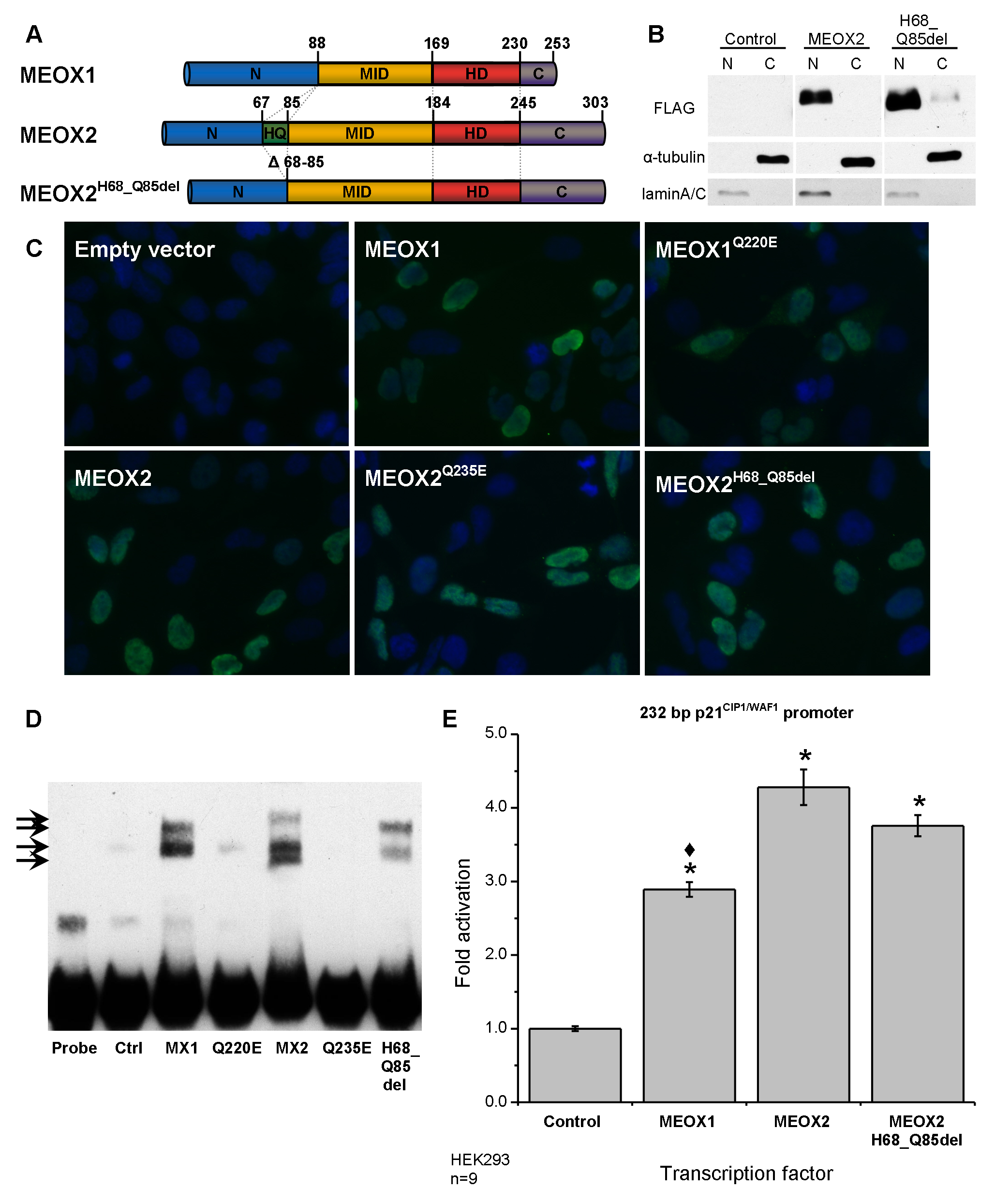

Supplement: Figure S5 — Deletion of the HQ rich domain of MEOX2 does not alter protein expression, localization or function. A) Schematic representation of the MEOX2H68_Q85del protein compared to wild-type MEOX1 and MEOX2. B) A representative western blot demonstrating the subcellular localization of MEOX2 H68_Q85del protein compared to wild-type MEOX2 in HEK293 cells, 48 hours after transfection. α-tubulin was used as a cytoplasmic (C) marker and lamin A/C was used as nuclear (N) marker. C) Representative fluorescent immunocytochemistry showing the localization and level of expression of the MEOX proteins in HEK293 cells 24 hours after transfection. The N-terminally tagged MEOX proteins were detected using an anti-FLAG antibody (green) and nuclei were stained with DAPI (blue). Empty vector was used as a negative control. D) Incubation of nuclear extracts from HEK293 cells transfected with MEOX1, MEOX2 or MEOX2H68_Q85del with the p21 probe resulted in the formation of distinct complexes (arrows). Homeodomain mutated MEOX1Q220E and MEOX2Q235E were unable to cause a specific shift of the DNA probe. E) MEOX2H68_Q85del activation of the luciferase reporter gene from the 232 bp p21CIP1/WAF1 promoter is comparable to wild type MEOX2. (TIF) [file pone.0029099.s005.tif]
