# Supplementary material for: Mechanisms of MEOX1 and MEOX2 Regulation of the Cyclin Dependent Kinase Inhibitors p21CIP1/WAF1 and p16INK4a in Vascular Endothelial Cells
Source: PLoS One. 2011 Dec 20;6(12):e29099. doi: 10.1371/journal.pone.0029099 (PMC3243699; doi:10.1371/journal.pone.0029099)
Supplement: Table S1 — List of PCR primers used to create MEOX1 and MEOX2 fusion proteins. (DOC) [file pone.0029099.s006.doc]

**Supplementary Table S1: List of PCR primers used to create MEOX1 and MEOX2 fusion proteins.**

| Primer | Direction | Sequence | Restriction site |
| --- | --- | --- | --- |
| MX003 | Forward | 5’-GCGAATTCGCAGTGGACAGCAGATGGACC-3’ | *EcoRI* |
| MX008 | Reverse | 5’-GGCTCGAGCTCTGAACTTGGAGAAGCTGC-3’ | *XhoI* |
| MX100 | Forward | 5’-GGGAATTCATGGATCCAGTGGCCAAC-3’ | *EcoRI* |
| MX095 | Reverse | 5’-GCCTCGAGTCACTCTGAACTTGGAGAAGC-3’ | *XhoI* |
| MX001 | Forward | 5’-GGGAATTCCCGGGATTATCCGAGCTCTG-3’ | *EcoRI* |
| MX005 | Reverse | 5’-GGGCTCGAGTAAGTGCGCATGCTCTGAG-3’ | *XhoI* |
| MX096 | Forward | 5’-GGGGATCCATGGAACACCCGCTCTTTGG-3’ | *BamHI* |
| MX097 | Reverse | 5’-GCCTCGAGTCATAAGTGCGCATGCTCTG-3’ | *XhoI* |
| MX022 | Forward | 5'-CCTTTCTGAGCGGCAGGTCAAAGTCTGGTTCGAAAAC  CGGAGGATG-3' |  |
| MX023 | Reverse | 5'-CATCCTCCGGTTTTCGAACCAGACTTTGACCTGCCGC  TCAGAAAGG-3' |  |
| MX024 | Forward | 5’-GACAGGTGAAAGTCTGGTTCGAAAACAGGCGGATGA  AGTGGAAGAGGG-3’ |  |
| MX025 | Reverse | 5’-CCCTCTTCCACTTCATCCGCCTGTTTTCGAACCAGACT  TTCACCTGTC-3’ |  |
| MX032 | Forward | 5’-CCCAGGAAAGAAAGGACAGCATTTACCGGTGGACAG  CAAGGAGCTGCGGC-3’ |  |
| MX108 | Forward | 5'-GTTTGCCAGCCAGCATCACAGGGGGGCTCTGCAAAC  CAACTGGCACCTCC-3' |  |
| MX109 | Reverse | 5'-GGAGGTGCCAGTTGGTTTGCAGAGCCCCCCTGTGATG  CTGGCTGGCAAAC-3' |  |
| NOTI | Reverse | 5’- GGGGCGGCCGCCTACTTATCGTCGTCATCCTTGTA-3’ | *NotI* |

Underlines indicate the restriction enzyme sequences.
