# Supplementary material for: Mechanisms of MEOX1 and MEOX2 Regulation of the Cyclin Dependent Kinase Inhibitors p21CIP1/WAF1 and p16INK4a in Vascular Endothelial Cells
Source: PLoS One. 2011 Dec 20;6(12):e29099. doi: 10.1371/journal.pone.0029099 (PMC3243699; doi:10.1371/journal.pone.0029099)
Supplement: Table S2 — List of PCR primers used to create the p21CIP1/WAF1 promoter luciferase constructs. (DOC) [file pone.0029099.s007.doc]

**Supplementary Table S2: List of PCR primers used to create the p21^CIP1/WAF1^ promoter luciferase constructs.**

| Primer | Direction | Promoter | Sequence | Restriction site |
| --- | --- | --- | --- | --- |
| MX062 | Forward | 849 bp | 5’-CCCCTCGAGGGCCAACAAAGCTGCTGCAAC  C-3’ | *XhoI* |
| MX068 | Forward | 505 bp | 5’-CCCTCGAGGGAAATTGCAGAGAGGTGCATC  GT-3’ | *XhoI* |
| MX069 | Forward | 426 bp | 5’-CCCTCGAGGCATTGGGTAAATCCTTGCCTGC  CA-3’ | *XhoI* |
| MX088 | Forward | 232 bp | 5’-CCCTCGAGCCCTCCTGCAGCACGCGAGGTTC  CG-3’ | *XhoI* |
| MX063 | Reverse | All | 5’-CCACAAGCTTCTGACTTCGGCAGCTGCTCAC-3’ | *HindIII* |

Underlines indicate the restriction enzyme sequences.
