# Supplementary material for: Mechanisms of MEOX1 and MEOX2 Regulation of the Cyclin Dependent Kinase Inhibitors p21CIP1/WAF1 and p16INK4a in Vascular Endothelial Cells
Source: PLoS One. 2011 Dec 20;6(12):e29099. doi: 10.1371/journal.pone.0029099 (PMC3243699; doi:10.1371/journal.pone.0029099)
Supplement: Table S3 — List of PCR primers used for qRT-PCR. (DOC) [file pone.0029099.s008.doc]

**Supplementary Table S3: List of PCR primers used for qRT-PCR.**

| Primer | Direction | Sequence |
| --- | --- | --- |
| P21 | Forward | 5’-GGAGACTCTCAGGGTCGAAAAC-3’ |
| P21 | Reverse | 5’-GGGCTTCCTCTTGGAGAAGATC-3’ |
| P16 | Forward | 5’-ATGGAGCCTTCGGCTGACT-3’ |
| P16 | Reverse | 5’-CGTAACTATTCGGTGCGTTG-3’ |
| β-ACT | Forward | 5’-AGGCCAACCGCGAGAAGATG-3’ |
| β-ACT | Reverse | 5’-CAGAGGCGTACAGGGATAGCAC-3’ |
