# Supplementary material for: Mechanisms of MEOX1 and MEOX2 Regulation of the Cyclin Dependent Kinase Inhibitors p21CIP1/WAF1 and p16INK4a in Vascular Endothelial Cells
Source: PLoS One. 2011 Dec 20;6(12):e29099. doi: 10.1371/journal.pone.0029099 (PMC3243699; doi:10.1371/journal.pone.0029099)
Supplement: Table S4 — List of EMSA probes. (DOC) [file pone.0029099.s009.doc]

**Supplementary Table S4: List of EMSA probes.**

| Probe | Gene | Binding site | Sequence |
| --- | --- | --- | --- |
| A6 | p21^CIP1/WAF1^ | Wild-type | 5’-CCCCGATGGCATTACAATTACAGATGACACT-3’ |
| MT11 | p21^CIP1/WAF1^ | Mutant | 5’-CCCCGATGGCA**G**TACAA**G**TACAGATGACACT-3’ |
| D16WT | p16^INK4a^ | Distal  Wild-type | 5’-CTCCCCCGTCCGTATTAAATAAACCTCATC-3’ |
| D16MT | p16^INK4a^ | Distal Mutant | 5’-CTCCCCCGTCCGTA**GG**AAATAAACCTCATC-3’ |
| P16WT | p16^INK4a^ | Proximal  Wild-type | 5’-GCTTTTTCTTATGATTAAAAGAAGAAGCCA-3’ |
| P16MT | p16^INK4a^ | Proximal Mutant | 5’-GCTTTTTCTTATGA**GG**AAAAGAAGAAGCCA-3’ |

Underlines indicate the homeodomain binding sites and bold letters indicate the mutant nucleotides.
