# Supplementary material for: Mechanisms of MEOX1 and MEOX2 Regulation of the Cyclin Dependent Kinase Inhibitors p21CIP1/WAF1 and p16INK4a in Vascular Endothelial Cells
Source: PLoS One. 2011 Dec 20;6(12):e29099. doi: 10.1371/journal.pone.0029099 (PMC3243699; doi:10.1371/journal.pone.0029099)
Supplement: Methods S1 — Supplementary methods. (DOC) [file pone.0029099.s010.doc]

**SUPPLEMENTARY METHODS**

**Calculation of MEOX protein homology**

The domains of human MEOX1 and MEOX2 proteins were compared using the BLASTP suite from NCBI by enabling the ‘align two or more sequences’ option [1]. Corresponding MEOX1/2 protein domains were aligned separately.

**MEOX1 and MEOX2 fusion protein expression constructs**

MEOX1 was PCR amplified using the MX003 and MX008 primers, digested with *EcoR*I/*XhoI* and then ligated into the pCMV-Tag4A vector (Stratagene), creating a C-terminal FLAG tagged construct. Subsequently, the MX100 and MX095 primers were used to amplify MEOX1 from the pCMV-Tag4A vector for cloning into the *EcoRI/XhoI* digested pCMV-Tag2B vector (Stratagene), to create an N-terminal FLAG tagged construct. MEOX2 was PCR amplified using the MX001 and MX005 primers, digested with *EcoRI/XhoI* and then inserted into the pCMV-Tag4A vector by ligation. Subsequently, the MX096 and MX097 primers were used to amplify MEOX2 from the pCMV-Tag4A vector for cloning into the *BamHI/XhoI* digested pCMV-Tag2B vector. MEOX1^Q220E^ was created using the pCMV-Tag4A-MEOX1 construct and the PCR mutagenesis primers MX022 and MX023. MEOX2^Q235E^ was created using the pCMV-Tag4A-MEOX2 construct and the PCR mutagenesis primers MX024 and MX025. MEOX2^K195_K245del^ was created from the pCMV-Tag4A-MEOX2 vector by splice overlap extension PCR using the MX032 primer with the MX001 and MX005 primers. MEOX2^H68_Q85del^ was created from the pCMV-Tag2B-MEOX2 vector by splice overlap extension PCR using the MX096, MX109, MX108 and MX097 primers. The MEOX1^Q220E^, MEOX2^Q235E^ and MEOX2^K195_K245del^ constructs were subsequently amplified from the pCMV-Tag4A vector using the MX096 and MX097 primers and cloned into the *BamHI/XhoI* digested pCMV-Tag2B vector. MEOX1, MEOX2 and MEOX2^Q235E^ were amplified from the pCVM-Tag4A vector using the MX3 (MEOX1) or MX1 (MEOX2) primer with the NOTI primer, digested with *EcoRI/NotI* and cloned into the pcDNA3.1 vector, thereby generating C-terminally FLAG tagged constructs in an alternate vector backbone.

**Luciferase assays**

Each well of HEK293 cells was transfected using 15 µL Lipofectamine 2000 Reagent (Invitrogen), 3 µg transcription factor vector DNA, 2 µg promoter vector DNA and 1 µg β-galactosidase vector DNA. For the p21^CIP1/WAF1^ promoter luciferase assays in HUVECs, cells were transfected with a total of 1 µg transcription factor vector DNA, 1 µg promoter vector DNA and 0.5 µg β-galactosidase vector DNA using 6.25 µL Lipofectamine LTX and 2.5 µL Plus Reagent (Invitrogen). For p16^INK4a^ promoter luciferase assays in HUVECs, cells were transfected with a total of 1 µg transcription factor vector DNA, 0.5 µg promoter vector DNA and 0.5 µg β-galactosidase vector DNA using 5 µL Lipofectamine 2000 (Invitrogen).

**Immunofluorescence**

Transduced HUVECs were washed once with phosphate-buffered saline (PBS) and then fixed in 4% paraformaldehyde (EMD Chemicals) for 30 minutes at room temperature. Cells on coverslips were washed three times with PBS then blocked with 5% goat serum (Sigma) in PBS containing 0.3% Triton-X 100 (PBS-T) overnight at 4°C. Primary mouse anti-FLAG antibody [M2] (Sigma) was diluted 1:1000 in blocking buffer and incubated with cells on coverslips for 2 hours at room temperature. Coverslips were then washed three times with PBS-T. Alexa Fluor 488 conjugated goat anti-mouse IgG secondary antibody (Invitrogen) was diluted 1:400 in blocking buffer and then incubated with cells on coverslips for 1 hour at room temperature. Coverslips were then washed three times with PBS-T and then incubated for 3 hours at room temperature with 1 mg/mL propidium iodide (Invitrogen) diluted 1:100 in blocking buffer. Coverslips were then washed 3 times with PBS-T and once with PBS prior to mounting onto slides using FluorSave Reagent (CalBiochem).

Alternatively, HEK293 cells (8×10^4^ cells/well) were plated onto collagen I (BD Biosciences) coated glass coverslips and then 48 hours later transfected with 4 μg MEOX expression vector DNA using 10 μL Lipofectamine 2000 Reagent (Invitrogen). Media was changed back to growth medium 4 hours post-transfection. Twenty four hours after transfection, cells were fixed and incubated with primary and secondary antibodies as described for transduced HUVECs**.** Coverslips were then washed three times with PBS-T and once with PBS prior to mounting onto slides using SlowFade Gold antifade reagent with DAPI (Invitrogen). Images were acquired with a ZeissAxioskop 2 mot plus microscope equipped with an AxioCam digital camera and AxioVision 4.6 software (Zeiss).

**Western blotting**

The mouse anti-FLAG [M2] primary antibody (Sigma) was diluted 1:5000 and incubated with the blot for 2 hours at room temperature. Primary mouse anti-p21^CIP1/WAF1^ antibody [CP74] (Sigma) was diluted 1:500 and then incubated overnight at 4°C and primary mouse anti-p16^INK4a^ antibody [DCS-50] (Santa Cruz) was diluted 1:50 and then incubated for 2.5 hours at room temperature. The loading controls, mouse anti-α-tubulin [DM1A] primary antibody (Abcam) was diluted 1:5000 and incubated for 2 hours at room temperature, while rabbit anti-actin primary antibody (Sigma) was diluted 1:2000 and incubated for 1 hour at room temperature. Horseradish peroxidase conjugated goat anti-mouse IgG and goat anti-rabbit IgG secondary antibodies (Molecular Probes) were diluted from 1:2500 to 1:10000 and incubated for 1 hour at room temperature. Nitrocellulose membrane with a pore size of 0.2 μM was used for p16^INK4A^/tubulin blots, while a 0.45 μM pore size was used for all other western blots. All antibodies were diluted in blocking buffer composed of 5% skim milk powder dissolved in Tris-buffered saline (TBS), with the exception of the p16^INK4a^ antibody which was diluted in 5% BSA (Sigma) dissolved in TBS. Antibodies were detected using Western Blotting Luminol Reagent (Santa Cruz Biotechnology) and a Fluor-S MAX MultiImager (Bio-Rad) or CL-Xposure blue X-ray film (Thermo Scientific). Quantity One software (Bio-Rad) was used to measure the relative band intensity (adj.vol. CNT*mm^2^). To control for loading irregularities, the intensity of the p21^CIP1/WAF1^ bands were normalized to those of actin, while the intensity of the p16^INK4a^ bands were normalized to those of tubulin.

**Recombinant protein production**

MEOX1 and MEOX2 coding sequences were excised from the pCMV-Tag2B vector with *EcoRI/XhoI* or *BamHI/XhoI*, respectively, and cloned into the pET-41a(+) vector (Novagen). Subsequently, these constructs were transformed into Rosetta-gami 2(DE3)pLysS competent cells (Novagen) for the production of recombinant proteins. Proteins were isolated using the BugBuster GST Bind Purification Kit (Novagen) as per the manufacturer’s recommendations with the addition of 0.5mM PMSF and protease inhibitor cocktail to all buffers.

**Electrophoretic mobility shift assays (EMSA)**

For cold competition reactions with the EMSA probe containing the proximal homeodomain binding sites from the p16^INK4a^ promoter, binding reactions containing 0.4 – 4 pmol unlabelled probe (10 – 100 molar excess) were incubated for 15 minutes at room temperature following which 40 fmol biotin end-labelled probe was added and incubated for an additional 30 minutes at room temperature.

A previously described probe sequence (A6) from the p21^CIP1/WAF1^ promoter, which contains MEOX2 binding sites, was used as a positive control [2]. For binding reactions, 200 ng of recombinant GST-fusion protein or 2.5 – 5 µL of HUVEC nuclear extract, and 20 fmol of biotin end-labelled probe were used. For cold competition reactions, 2 – 10 pmol unlabelled probe was added (100 – 500 molar excess) prior to the addition of the biotin labelled probe. Super-shift reactions contained 1 µg normal mouse IgG (Millipore) or anti-FLAG [M2] antibody (Sigma). All incubations were carried out as described for the p16^INK4a^ promoter probes. Luminescence was detected using a Fluor-S MAX MultiImager (Bio-Rad) or CL-Xposure blue X-ray film (Thermo Scientific).

HEK293 cells were plated and transfected as described for western blotting. Nuclear proteins were isolated using the NE-PER nuclear and cytoplasmic extraction kit (Pierce). Binding reactions containing 1-3 µL nuclear extract and 20 fmol of biotin end-labelled probe were incubated for 30 minutes at room temperature.

**REFERENCES**

1. Altschul SF, Madden TL, Schaffer AA, Zhang J, Zhang Z, et al. (1997) Gapped BLAST and PSI-BLAST: a new generation of protein database search programs. Nucleic Acids Res 25: 3389-3402.

2. Chen Y, Leal AD, Patel S, Gorski DH (2007) The homeobox gene GAX activates p21WAF1/CIP1 expression in vascular endothelial cells through direct interaction with upstream AT-rich sequences. J Biol Chem 282: 507-517.
